# Effect of vacancies and edges in promoting water chemisorption on titanium-based MXenes

**DOI:** 10.1186/s40580-023-00364-8

**Published:** 2023-04-01

**Authors:** Edoardo Marquis, Francesca Benini, Babak Anasori, Andreas Rosenkranz, Maria Clelia Righi

**Affiliations:** 1 Department of Physics and Astronomy, Alma Mater Studiorum – University of Bologna, Viale Berti Pichat 6/2, 40127 Bologna, Italy; 2 Department of Mechanical and Energy Engineering, Integrated Nanosystems Development Institute, Indiana University-Purdue University Indianapolis, Indianapolis, IN 46202 USA; 3 School of Materials Engineering, Purdue University, West Lafayette, IN 47907 USA; 4 Department of Chemical Engineering, Biotechnology and Materials, University of Chile, Avenida Beaucheff 851, 8370456 Santiago de Chile, Chile

**Keywords:** MXenes, DFT, Water chemisorption, Hydrophilicity, Oxidation

## Abstract

The functionality of two-dimensional (2D) transition metal carbides and nitrides (MXenes) in technological applications greatly depends on their wettability. For instance, MXenes’ layer stability against degradative oxidation is notably reduced when stored in aqueous solutions, leading to the transformation into oxides. In this work, we study water adsorption on Ti-based MXenes by ab initio calculations. The energy gains for the molecular adsorption on Ti_*n*+1_X_*n*_T_2_ is evaluated as a function of the termination (T = F, O, OH, mixture), the carbon/nitrogen ratio (X = C, N), the layer thickness (*n*) and water coverage. MXenes’ hydrophilicity tends to increase due to the presence of defects as vacancies and flake edges. We demonstrate that physical adsorption occurs through hydrogen bonding on both defect-free layers and layers containing C/N or Ti atomic vacancies, with –OH terminations providing the strongest interactions (0.40–0.65 eV). In contrast, strong water chemisorption is observed on surfaces with a single termination vacancy (0.60–1.20 eV), edges (0.75–0.85 eV), and clusters of defects (1.00–1.80 eV). We verified that the presence of undercoordinated Ti atoms on the surface is the key factor in promoting H_2_O chemisorption, i.e., the degradative oxidation.

**Graphical Abstract:**

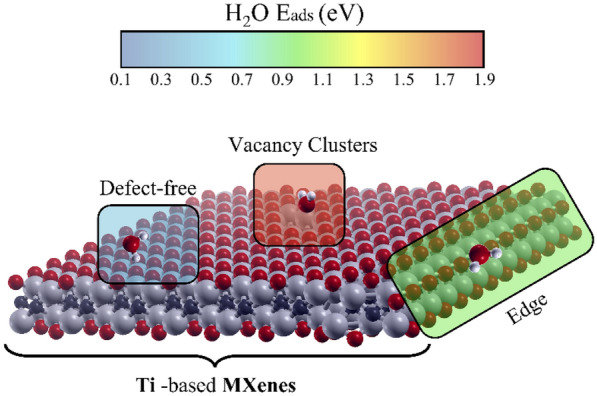

**Supplementary Information:**

The online version contains supplementary material available at 10.1186/s40580-023-00364-8.

## Introduction

Two-dimensional (2D) transition metal carbides, nitrides and carbonitrides (MXenes) are among the most studied 2D materials today owing to their wide technological applicability [1, 2]. They are conventionally described by the chemical formula M_*n*+1_X_*n*_T_*x*_, for which M represents an early transition metal (Ti, V, Cr, Mo, Nb, etc.), X is carbon or/and nitrogen, T_*x*_ is the number (*x*) and type (T) of surface terminations, while *n* stands for the layer thickness (*n* = 1–4) [3–5]. By varying MXenes’ synthesis and composition, their physico-chemical properties can be intentionally designed. After MXenes’ first synthesis in 2011 [6], several applications have been proposed and explored, such as energy conversion and storage [7, 8], sensors [9, 10], electromagnetic interference shielding [11, 12], catalysis [13–15] and tribology [16, 17].

For many applications, the precise understanding of the wettability of 2D materials is mandatory to improve their functionality. It is known that the properties of 2D materials depend on environmental conditions [18, 19]. For instance, the presence of water promotes low-friction sliding between graphene layers, while it worsens the tribological performance of molybdenum disulfide (MoS_2_) [20, 21]. The influence of humidity on the tribological behavior has been also investigated for Ti_3_C_2_T_*x*_ multi-layer coatings by Marian et al., finding that the friction and wear performance is detrimental for higher relative humidities [22]. It is well accepted that MXenes show an intrinsic hydrophilicity, mainly attributable to the presence of hydroxyl, oxygen and fluorine terminations [3]. However, the hydrophilicity of this family of 2D materials has been scarcely investigated with both experimental and computational approaches [23–25]. Contact angle measurements reveal that the wettability of MXenes is not homogeneous but can vary depending on local surface features as well as the presence of contaminations [23]. Apart from these general conclusions, a complete characterization of MXenes’ hydrophilicity is lacking.

Another important issue concerning the interaction of MXenes with water relates to their degradative oxidation. This phenomenon, spontaneously occurring under ambient conditions, inevitably compromises the synthesis and subsequent applicability of MXenes [26, 27]. Many experiments and calculations have confirmed the intrinsic tendency of MXenes to transform into oxides, while light and temperature tend to accelerate this process [25, 28–34]. Several factors affecting the oxidation rate of MXenes have been investigated, such as layer microstructure and atmosphere/solvent composition [26]. One of the critical factors in stability of MXenes is to know which oxidant species (O_2_ or H_2_O) is mainly responsible for the degradative process. In earlier studies, oxygen dissolved in MXenes’ colloidal solutions was thought to play the major role [33]. However, recent studies on the oxidation kinetics performed by ultraviolet–visible and Raman spectroscopy revealed that water seems to be the key component leading to degradation [35]. Recently, atomistic insights on MXenes’ oxidation in aqueous solutions were provided by means of first principle molecular dynamics (MD) simulations [25]. Their results strongly support the experimental observation that water is an oxidant, which is strong enough to attack and degrade MXenes.

In this context, fundamental aspects regarding the interaction of MXenes with water need to be unraveled. The characterization of hydrophilicity for different sites, both defective and not, can be a starting point for further investigations. In this work, we exploit Density Functional Theory (DFT) calculations to study the interaction of H_2_O with Ti-based MXenes. We mapped the hydrophilicity of defect-free surfaces by varying the termination type (T), the carbon/nitrogen ratio (X), as well as the layer thickness (*n*). The analysis is repeated for defective MXene flakes, as they are known for being prone to oxidation [36–38]. The role of single atomic vacancies, cluster of defects, and edges in promoting the chemisorption of water is addressed and explained, focusing on both energetical and structural aspects. Since MXene surfaces are usually terminated with a random distribution of –F, –O and –OH groups [38–41], we also map the hydrophilic areas for mixed terminated MXenes. Lastly, we discuss the influence of increasing water coverage on the adsorption process.

## Systems and methods

The analysis was performed by means of spin-polarized DFT calculations as implemented in the version 6.7 of the Quantum ESPRESSO suite [42–44]. We used the generalized gradient approximation (GGA) within the Perdew–Burke–Ernzerhof (PBE) parametrization to describe the exchange-correlation functional [45]. To properly consider the van der Waals interactions, we used an ad-hoc modification of the Grimme D2 dispersion correction scheme (i.e., D_NG_) [46, 47], which differs from the standard D2 only for the C_6_ coefficient and the van der Waals radius R_0_ of the Ti atoms, which are replaced with those of Ar, i.e. the preceding noble gas in the periodic table (hence the acronym NG). In a previous work, [46] we demonstrated that the use of PBE + D_NG_ is more accurate in capturing long-range van der Waals interactions than other dispersion-corrected DFT functionals (e.g. vdW-DF2) when MXenes are involved. The electronic wave-function was expanded on a plane-wave basis, truncated with a cutoff of 50 Ry, while a cutoff of 400 Ry was applied for the charge density, in agreement with our previous study on Ti-based MXenes [46]. The ionic species were described by ultrasoft pseudopotentials. The structural relaxations were carried out using convergence thresholds of 10^−4^ Ry and 10^−3^ Ry/Bohr for the total energy and the atomic force components, respectively, while Gaussian smearing of 0.02 Ry was used to better describe the electronic states occupation around the Fermi level. Additional information on convergence tests is shown in Additional file 1: Fig. S1.

To model MXene surfaces, we considered 4 × 4 orthorhombic supercells, ensuring at least 15 Å of vacuum between periodic replicas along the *z* direction. To model MXene edges, the *b* lattice parameter of the supercell was doubled, leading to distances between ribbon replicas of at least 10 Å along the *y* direction. For all surfaces, the sampling of the Brillouin zone was done with a 3 × 4 × 1 *k*-points Monkhorst-Pack grid, while an equivalent reduced 3 × 2 × 1 grid was used for the ribbons [48]. To model the isolated water, we employed a cubic cell with side of about 25 Å, which is large enough to consider the molecule as isolated. More details on the supercells are provided in Additional file 1: Figs. S2, S3.

**Fig. 1:**
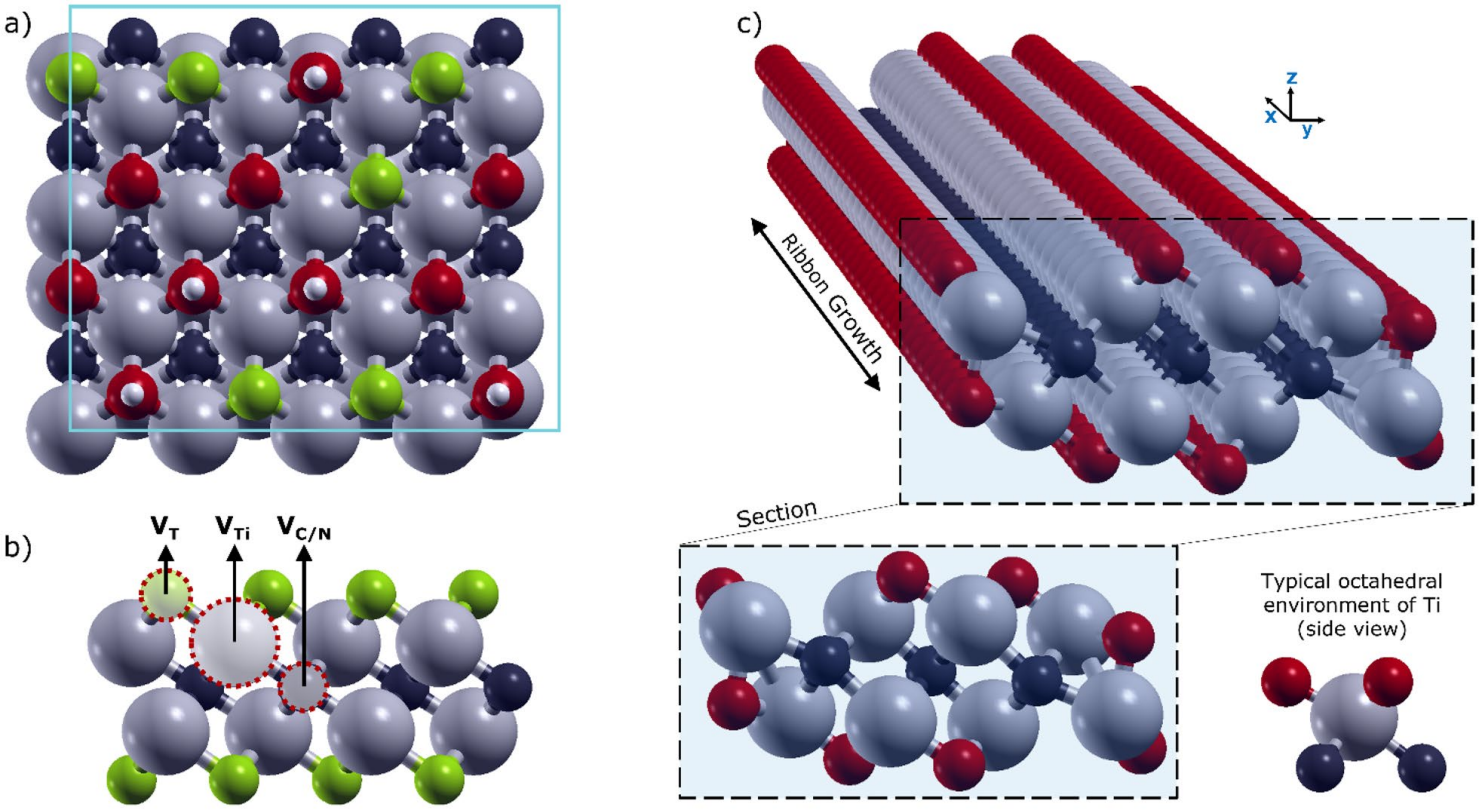
**a** Top-view of the 4 × 4 orthorhombic cell employed for the mixed terminated MXene surface. The supercell is also representative of homogeneously terminated surfaces. **b** Schematic of the single atomic defects considered: termination (V_T_), titanium (V_Ti_) and carbon/nitrogen (V_C/N_) vacancies. **c** Perspective view (above) and cross-section (below) of the Ti_8_C_3_O_8_ nanoribbon, that we used for the edge effect oxidation. In the lower right corner, we provided a schematic of the octahedral coordination usually found for Ti atoms in MXenes, which instead is distorted in nanoribbons. Chemical elements are represented by different colors: Ti-grey, C-black, F-green, O-red, H-white

An exemplary selection of the MXene models employed is depicted in Fig. 1. First, we considered eight orthorhombic defect-free surfaces: six layers having the general formula Ti_2_XT_2_, with X = C or N, and T = F, O or OH, one thicker layer with formula Ti_4_C_3_F_2_, and a mixed-terminated MXene surface. For the latter case, the 4 × 4 orthorhombic model allowed us to simultaneously consider 16 surface groups, that we randomly assigned to be 6 –F, 5 –O and 5 –OH groups (Fig. 1a). Then, by removing a termination (V_T_), a titanium atom (V_Ti_), or a carbon/nitrogen atom (V_X_), we introduced a single-atom defect, as schematically reported in Fig. 1b. We modelled a vacancy concentration of 6.25%, obtained by the removal of one atom from the employed 4 × 4 supercell. This value is compatible with data on Ti_3_C_2_T_x_ monolayers obtained by scanning transmission electron microscopy (STEM) measurements that suggest a large range of defect densities depending on the synthesis conditions as well as the quality of the initial bulk precursor [49, 50]. Indeed, single-atom defects are commonly found in MXenes synthesized with low etchant concentrations. Vacancy clusters are often detected for harsher synthesis conditions (e.g., high hydrofluoric acid concentrations).

We compared our models with those of Gouveia et al. [51], finding an excellent agreement in terms of vacancy formation energy and structural features. Layers with clusters of vacancies were modelled by removing at least two adjacent atoms from the surface. For instance, a “*p*V_Ti_ + *q*V_T_” cluster is obtained by removing *p* atoms of Ti and *q* termination groups (*p* = 1, 2 and *q* = 1, 2, 3). MXene nanoribbons were built according to the work of Zhao et al. [52], in which the stability of different edge reconstructions for Ti_2_CT_2_ (with T = F, O or OH) was investigated by means of DFT. We focused on those zigzag nanoribbons having the empirical formula Ti_8_C_3_T_8_ that showed the lowest edge energy. To model the analogous nitrogen-based ribbons, i.e., Ti_8_N_3_T_8_, we kept the same geometrical structure, allowing the relaxation of the atomic positions. In Fig. 1c, we exemplarily present a perspective and a sectional view of the Ti_8_C_3_O_8_ nanoribbon, with the outermost Ti atoms that have lost their typical octahedral geometry in favor of a distorted tetrahedral coordination.

The partial atomic charges were evaluated by means of the Bader Charge analysis [53–56]. The adsorption energy (E_ads_) is calculated as the difference between the total energy of the interacting system and the sum of the energies of the substrate and the isolated water molecule. To study the dependence of E_ads_ on the water coverage, we normalized the energy value by the number *n* of adsorbed water molecules:

1 $${\text{E}}_{\text{ads}}\left(n\right)=\frac{{\text{E}}_{\text{total}}-(n\cdot {\text{E}}_{{\text{H}}_{2}\text{O}}+{\text{E}}_{\text{substrate}})}{n}$$

We will refer to the energy gain (E_gain_) as the absolute value of the adsorption energy (i.e., E_gain_ = |E_ads_|).

To increase the probability of finding the lowest energy configuration for the adsorbed water, we explored different starting molecular configurations as reported in Additional file 1: Fig. S4. They differ regarding the H_2_O orientation relative to the surface (in-plane water, oxygen-up and oxygen-down), the H_2_O rotation angle with respect to the perpendicular axis and the lateral position. Despite a large number of initial configurations, the final geometries were often equivalent, coinciding with local minima or global minimum.

## Results and discussion

We have divided our results and discussion into seven sections. The first section holistically summarizes the energetics calculated for all systems considered in this work. In the subsequent Sect. 3.2–3.5, the physical interpretation for the configurational aspects of the MXene–water interaction is presented for each type of surface. Finally, we present the hydrophilicity map for a mixed terminated MXene (Sect. 3.6) and the influence of the water coverage on the average adsorption energy (Sect. 3.7).

### Hydrophilicity of MXenes

**Fig. 2:**
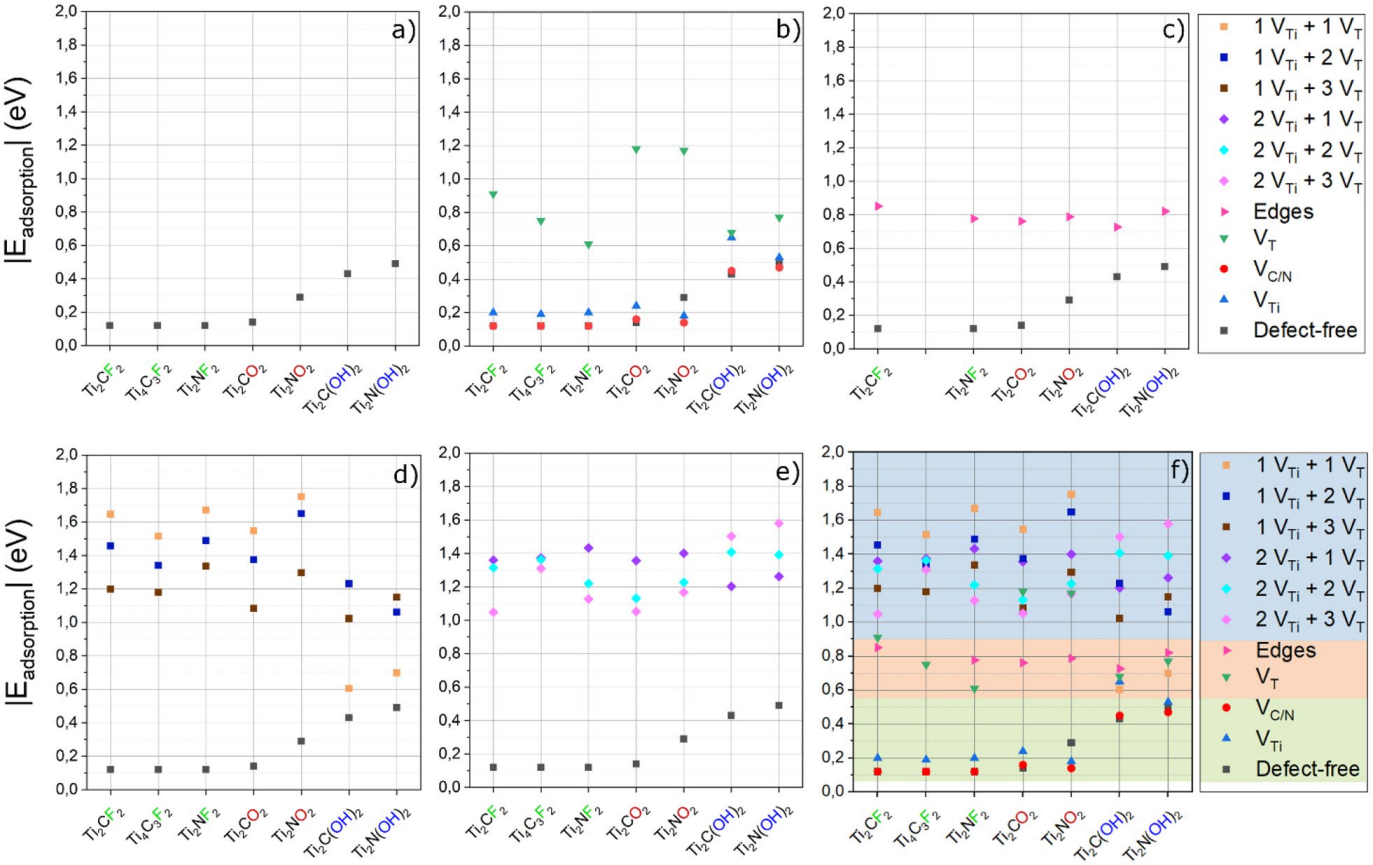
Absolute values of the adsorption energy of one single water molecule on different types of MXenes: **a** defect-free surfaces, **b** surfaces with single vacancy of C/N (V_C/N_), Ti (V_Ti_) or termination (V_T_), **c** edges, **d**, **e** surfaces with cluster of defects (*p*V_Ti_ + *q*V_T_), consisting of *p* Ti vacancies and *q* termination vacancies. **f** Summarizes all cases considered. The highlighted intervals reflect the extent of the interaction with water: blue stands for strong chemisorption and green reflects physisorption, while red points to an intermediate behavior

The hydrophilic character of a material at the atomistic level can be related to the energy gain associated with the adsorption of water molecules. Energy gains related to the adsorption of a single water molecule are displayed for the defect-free (Fig. 2a) and single vacancy-containing MXenes (Fig. 2b). We studied Ti_2_XT_2_ as the example MXene and investigated the effect of the surface termination (T = F, O and OH), carbide vs. nitride (X = C and N), and the presence of vacancy on the metal site (V_Ti_), on the X site (V_C/N_) and the surface termination sites (V_T_). We also studied MXenes with a higher thickness (i.e., Ti_4_C_3_F_2_) to a limited extend, due to the increased computational effort required for simulating these larger systems. However, it is known that adsorption phenomena mainly affect the atoms closest to the surface, which agrees with our findings. Therefore, no remarkable differences were found when switching from a thinner MXene (Ti_2_CF_2_) to a thicker analogous (Ti_4_C_3_F_2_).

The fully terminated surfaces interact with water via hydrogen bonds. Fluorine and oxygen terminations can act as weak H-bond acceptors, while hydroxyls can also play the role of the donor. This behavior leads to an increase in the energy gain by moving from –F and –O (0.12–0.29 eV) to –OH (0.43–0.49 eV) terminated surfaces (Fig. 2a). In any case, adsorption energies of H_2_O are greater on MXene monolayers compared to other 2D materials such as graphene or MoS_2_ (0.12–0.15 eV) [57], demonstrating their intrinsic hydrophilic behavior.

In general, the removal of an atom from a surface (i.e., single vacancy) was supposed to enhance its reactivity towards water adsorption [58]. However, for the internal MXene vacancies, including C/N vacancies (V_C/N_ shown as red circles) and Ti vacancies (V_Ti_, shown as blue triangles), the interaction with H_2_O appears to be almost the same as for the defect-free MXenes (Fig. 2b). The negligible change in energy could be explained by the fact that the water molecule is not small enough to approach these internal defects, resulting in negligible stabilization. In the case of termination vacancies (V_T_, as shown as green triangles), the dangling bonds of the underlying Ti atoms make the surface extremely reactive. The oxygen atom of H_2_O can physically fill the hole left by the missing T_*x*_, and due to its two lone pairs, it can provide stability to the undercoordinated Ti atoms. For the T_*x*_-vacancy, the associated energy gains range from 0.61 to 1.18 eV, depending on the reactivity of the MXene (Fig. 2b). It is worth noting that the water adsorption energy on MXene surfaces with Tx-vacancy is comparable to that of titanium dioxide (TiO_2_) reported in literature. The most common polymorphs of TiO_2_, anatase and rutile, are both composed of distorted TiO_6_ octahedra, with the Ti atoms showing octahedral coordination similar to MXene layers. As reported in Ref. [59], water interacts with the anatase (101) and rutile (110) crystal faces through the coordination of the water oxygen atom to the outermost Ti atoms. Indeed, the exposed Ti atoms are undercoordinated (fivefold-coordinated), thus prone to being stabilized by the oxygen atom of water. Interestingly, based on DFT calculations [60, 61], the water adsorption on the (101) crystal face of anatase and (110) face of rutile is found to be 0.84 and 0.94 eV, respectively, which is within the E_ads_ energy range observed for MXene’ surfaces with defects.

As the next step in our analysis, we considered the effect of MXene edges as the defects. Figure 2c refers to the water interaction with MXene edges showing the lowest formation energy [52]. At the MXene edges, the outermost Ti atoms have lost their typical octahedral geometry, which become defects sites that can interact with H_2_O (see Sect. 3.4). Because the edges are mainly undercoordinated Ti atoms, the strength of the interaction is almost independent of the MXene composition, ranging between 0.73 and 0.85 eV (red triangles in Fig. 2c). This finding agrees with the fact that in almost all MXenes, 2D flakes’ edge oxidation can be observed during storage regardless of the flake composition and the number of transition metal layers (*n*) in MXene, and edge capping has been utilized as a way to slow down the oxidation [62, 63].

Finally, the energy gains concerning H_2_O interacting with a cluster of defects are presented in Fig. 2d, e. We explored a wide range of 2D flakes consisting of a single (or double) Ti vacancy and one, two and three termination vacancies. Generally, all energy gains are greater than 1.00 eV, suggesting the strong chemisorption of the water. Some exceptions are found for those layers functionalized with –OH with one Ti vacancy (Fig. 2d), that are not reactive enough to promote the chemisorption of water. This peculiar deviation between –OH and –F/–O terminated MXene flakes will be addressed in detail in the following sections.

Figure 2f shows a collective representation of the water adsorption energies on MXene flakes from defect-free to vacancy clusters. We divided the energy values into three regions, according to the nature and strength of the molecule-surface interaction. The green region ranging between 0.10 and 0.55 eV includes pure physisorption of water via hydrogen bonds. For E_ads_ greater than 0.60 eV, the oxygen of water establishes chemical bond(s) with the undercoordinated Ti atom(s) of the surface, leading to chemisorption. We distinguished a weak chemisorption, 0.6 eV < E_ads_ < 1.0 eV (red region), from a stronger chemisorption, E_ads_ above 1.00 eV (blue region). Defect-free MXenes and single vacancies of Ti and C/N (in green) lead to adsorption energies that are mainly affected by the type of termination (T). For these surfaces, the interaction with water does not exceed 0.55 eV (green region). Intermediate energy values (0.60–0.90 eV) are mainly found for water on single termination vacancies and edges. In contrast, the energy range associated with clusters of vacancies (0.90–1.80 eV), highlighted in blue, is the highest among all and compatible with strong chemisorption of water (i.e., shorter Ti–OH_2_ bond distances). Figure 2f identifies defect clusters as the sites interacting more strongly with the H_2_O molecule irrespective of the termination type and the carbon/nitrogen content as discussed in Sect. 3.5.

### Water adsorption on defect-free surfaces

**Fig. 3:**
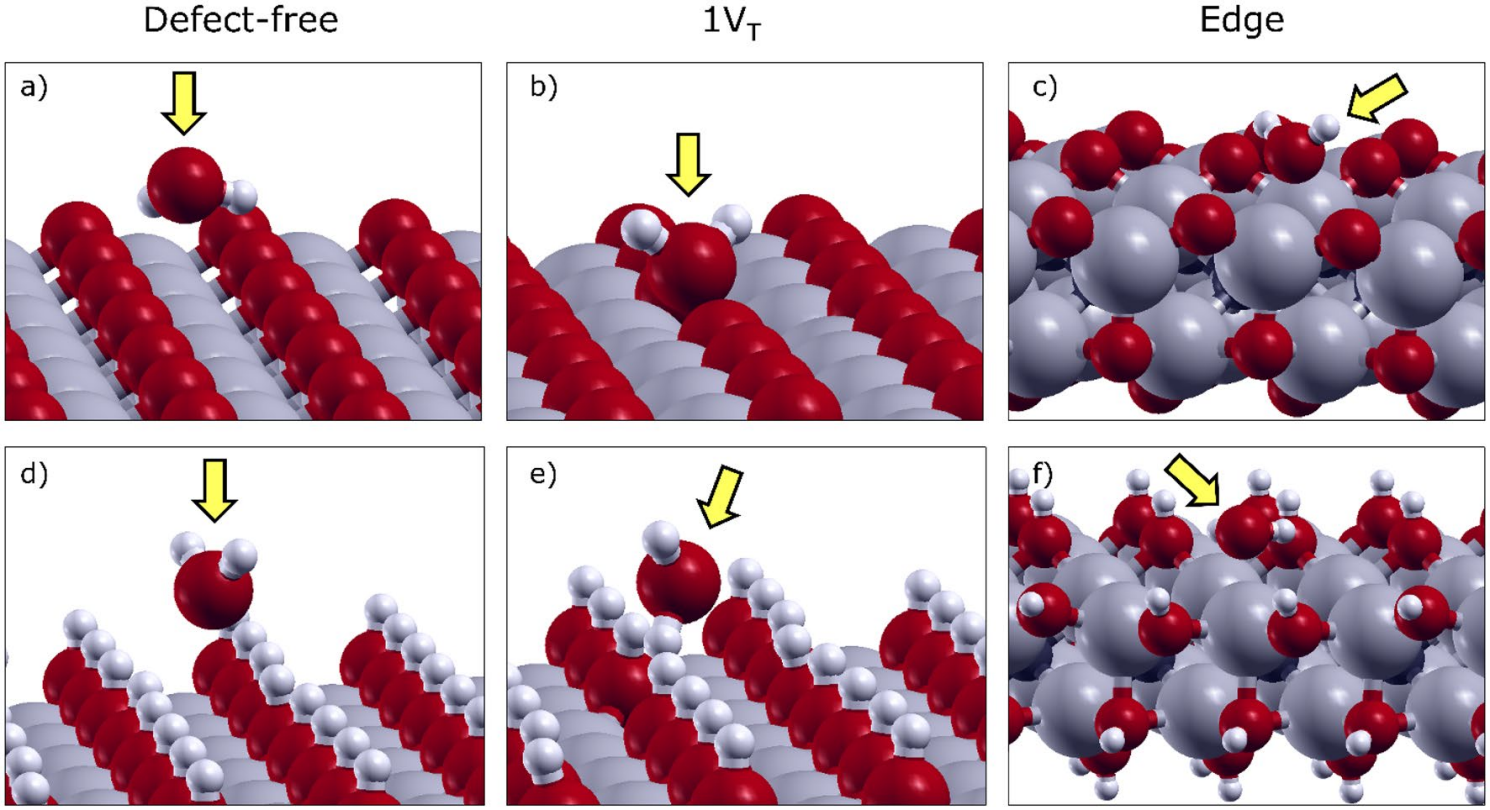
Perspective view of the optimized configuration of H_2_O interacting with (**a**, **d**) defect-free MXenes, (**b**, **e**) surfaces with a vacancy of termination (V_T_), and (**c**, **f**) edge of ribbons. Ti_2_CO_2_ (above) and Ti_2_C(OH)_2_ (below) are taken as examples. The yellow arrow highlights the presence of the water molecule. Ti atoms are shown in gray, O in red, C in black and H in white

The optimized adsorption configurations of water on defect-free surfaces are illustrated for Ti_2_CO_2_ and Ti_2_C(OH)_2_ in Fig. 3a, d. For these MXene surfaces, the role of layer thickness and carbon/nitrogen content are negligible compared to the effect induced by the termination. In particular, –F and –O behave similarly, acting as weak acceptors of hydrogen bonds. Due to the electrostatic repulsion between the passivating groups and the oxygen of water, only a slight approach is allowed (Fig. 3a). Therefore, long-distance H-bonds are established (the distances between H_2_O and –F and –O terminations are in the range 2.34–2.43 Å), resulting in weak interactions. In contrast, –OH terminations (Fig. 3d) can act as donors of hydrogen bonds, leading to shorter distances (1.97–2.03 Å) and stronger interactions.

### Water adsorption on surfaces with single-atom defects

Single vacancy sites were modeled by removing the atoms closest to the surface, in three types, V_T_, V_Ti_ and V_C/N_ as a surface with a vacancy of termination, titanium, and carbon/nitrogen, respectively. In Fig. 3b, e, we shown the optimized configurations of the water molecule on Ti_2_CO_2_ and Ti_2_C(OH)_2_ surfaces with a missing termination (V_T_). Configurations concerning other vacancy sites (i.e., V_Ti_ and V_C/N_) are provided in Additional file 1: Fig. S5. Generally, the same optimized orientation of H_2_O was found when interacting with F- and O-terminated nitrides and carbides. This characteristic probably relates to the similar physical-chemical properties (e.g., high electronegativity, H-bonds acceptor capability) of both terminations. Moreover, OH-terminated carbides and nitrides retain a similar behavior to each other as well.

Neither V_C/N_ nor V_Ti_ affects the interaction with water compared to defect-free surfaces. Once again, the formation of hydrogen bonds is the main driving force that regulates the interaction. However, the optimal H-bond distance was found to vary on defective surfaces and followed a specific trend. Taking Ti_2_NF_2_ as an example, the average H-bond distance is 2.37 Å, 2.33 Å and 2.20 Å for a defect-free surface, V_C/N_ and V_Ti_, respectively.

The reactivity towards water can be notably increased in the presence of surface termination vacancies (V_T_), favoring the transition from physisorption to chemisorption (green triangles in Fig. 2b). The absence of one surface termination causes undercoordination of the three surrounding Ti atoms. Some of these dangling bonds can be saturated by the lone pairs of the oxygen atom of water. However, only for surfaces terminated with –F and –O, water can approach the Ti-atoms (Fig. 3b). In contrast, for surfaces with a single OH-vacancy in Ti_2_N(OH)_2_ and Ti_2_C(OH)_2_, hydroxyl groups close to the V_T_ defect tend to trap H_2_O into a H-bond network, before reaching the chemisorption site (Fig. 3e). The chemisorption values vary between 0.61 and 1.18 eV for F- and O-terminated surfaces, while the absolute values depend on various factors. We identified three main contributions to this variation: (i) the residual positive atomic charge of the undercoordinated Ti atoms, (ii) the equilibrium distance between the oxygen atom of water and titanium, (iii) the number of Ti atoms stabilized by the water chemisorption. For instance, the energy gains obtained on V_T_ substrates for Ti_2_NF_2_ (0.61 eV), Ti_2_CF_2_ (0.91 eV) and Ti_2_NO_2_ (1.17 eV) show a correlation with the partial atomic charges of the Ti atoms surrounding the vacancy (q_Ti_ = + 1.45*e*, + 1.53*e*, + 1.65*e*, respectively). Although the high value of q_Ti_ found on Ti_4_C_3_F_2_ (+ 1.60*e*) and Ti_2_CO_2_ (+ 1.79*e*), for these two MXenes the oxygen atom of H_2_O was found to stabilize only one titanium, instead of two. This compensation leads to the energy fluctuation trend shown in Fig. 2b.

### Chemisorption on edges

To study the effect of MXene flake edges, we focused on MXene ribbons (1D MXenes). Different reactive sites appear on the edges, depending on the specific cut and reconstruction. However, based on a study on MXene nanoribbons [49], we decided to restrict our study to those nanoribbons that have the lowest formation energy, thus showing the highest stability. Therefore, the adsorption values collected in Fig. 2c, ranging between 0.73 and 0.85 eV, can be considered as the lower limit that can easily increase in presence of more unstable edges. Representations of the optimized Ti_8_X_3_T_8_ nanoribbons (X = C or N, and T = F, O or OH) are presented in Additional file 1: Fig. S6. The main geometrical feature of the MXene edge (nanoribbons) is a distortion of the chemical bonds between Ti atoms and surface terminations. At the edge, the classical octahedral geometry is lost for the outermost Ti atoms, leading to an unconventional distorted tetrahedral coordination (Fig. 1c).

The configurations of water interacting with MXene edges (1D-MXenes Ti_8_C_3_O_8_ and Ti_8_C_3_(OH)_8_) are provided in Fig. 3c, f. The defective undercoordinated Ti atom along the edges constitutes the most reactive site to interact with water. Regardless of the termination type and C/N content, the oxygen of water interacts with one or two undercoordinated Ti atom(s), similarly to the MXene flake with a termination vacancy (V_T_). We found that the energy gain mainly depends on the H_2_O–Ti distance, the number of Ti atoms involved, and the H_2_O-assisted reconstruction of the edge. In most cases (including Fig. 3f), water interacts with a single titanium atom, with a narrow H_2_O–Ti distances distribution (2.25−2.31 Å). In other cases (Fig. 3c), H_2_O prefers to interact with two Ti atoms, leading to an increase in the average distance. A visible reconstruction of the edge is observed only for Ti_8_C_3_F_8_, where a –F termination moves inwards, giving way to the oxygen of water to interact with the edge (Additional file 1: Fig. S6). For other systems, only minor displacements can be seen, such as slight rotations of –OH groups.

### Chemisorption on vacancy clusters

**Fig. 4:**
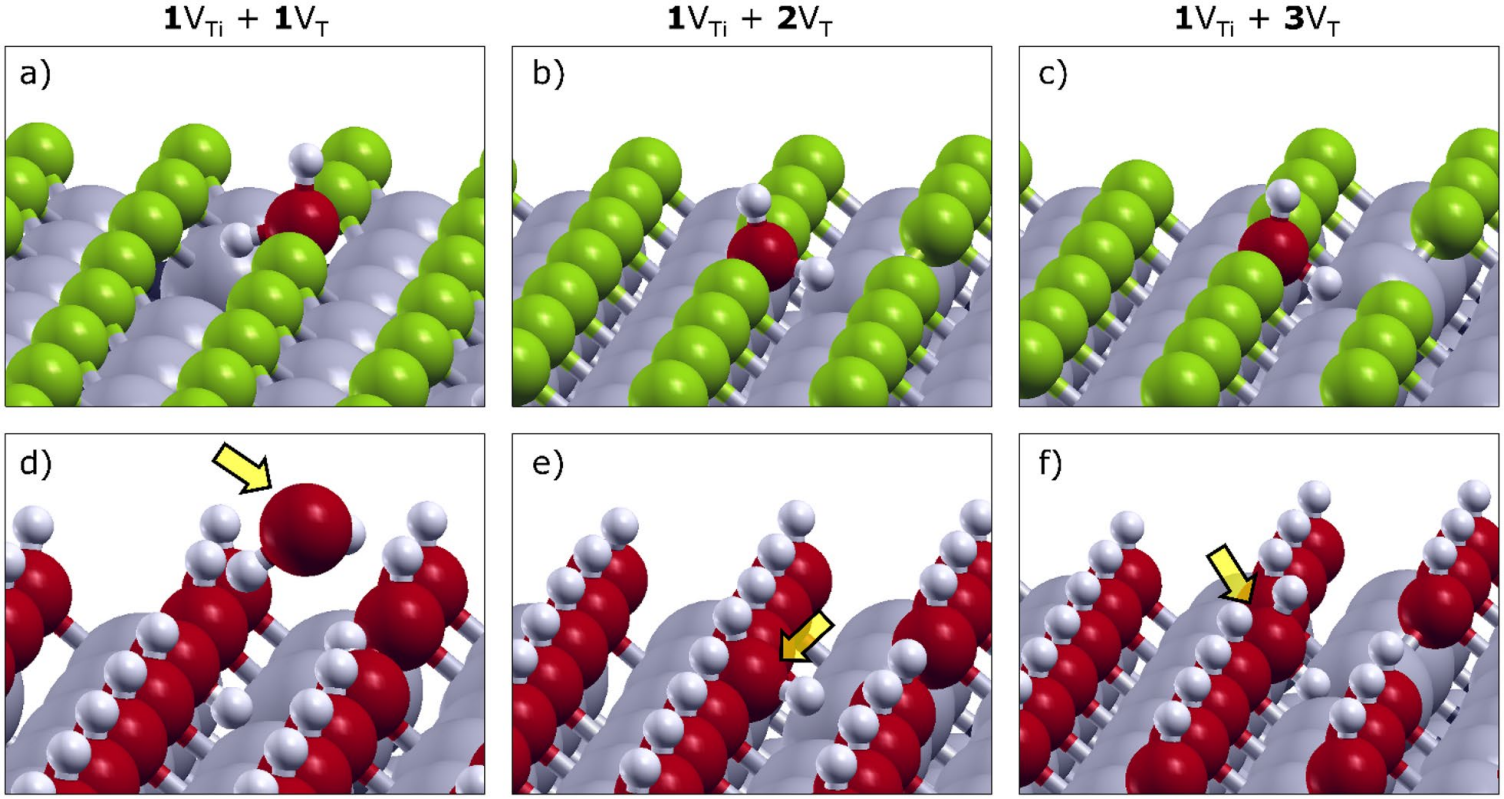
Optimized configurations of H_2_O adsorbed on different clusters of vacancies for Ti_2_CF_2_ (**a**–**c**) and Ti_2_C(OH)_2_ (**d**–**f**). The clusters of defects consist of one titanium vacancy and up to three termination vacancies, as 1V_Ti_ + 1V_T_ (**a**, **d**), 1V_Ti_ + 2V_T_ (**b**, **e**) and 1V_Ti_ + 3V_T_ (**c**, **f**). The yellow arrow highlights the presence of the water molecule interacting with the defected Ti_2_C(OH)_2_ surface

Various combinations of titanium (V_Ti_) and termination (V_T_) vacancies were considered as defect clusters (*p*V_Ti_ + *q*V_T_), exploring *p* = 1, 2 and *q* = 1, 2, 3. In Fig. 4, we only focus on single Ti vacancy cases (*p* = 1) with vacancy clusters on surface terminations (*q* = 1, 2, 3), providing the optimized configurations for two representative materials, i.e., Ti_2_CF_2_ and Ti_2_C(OH)_2_. In Additional file 1, the configurations of water adsorbed on 2V_Ti_ + *q*V_T_ clusters are also shown and discussed.

For vacancy clusters (1V_Ti_ + *q*V_T_) on O- and F-terminated MXenes (Fig. 4a–c), the H_2_O molecule provides two strong stabilizing effects. First, the oxygen atom of water saturates a V_T_, interacting with the remaining two surrounding Ti atoms. Moreover, one H of the water can point towards the hole left by V_Ti_. With the lack of a titanium atom, many T and C/N atoms become undercoordinated, leading to a densification of the negative charge centered on the V_Ti_ defect. This excess of charge density is mitigated when one of the two positively charged hydrogens of H_2_O is pointed towards the hole. It is worth noting that the excess of charge density is reduced with an increasing number of V_T_. In other words, when a V_Ti_ is formed, the more neighboring terminations are missing, the less reactive the surface will be.

For OH-terminated MXenes (Fig. 4d–f), similar configurations are found, except for 1V_Ti_ + 1V_T_ cluster. This peculiar defect (Fig. 4d) is already stabilized without the necessity of interacting with the water molecule. Due to the lack of a titanium atom, the surrounding hydroxyl terminations can rotate and point the hydrogens towards the hole. When the number of terminations surrounding the V_Ti_ is reduced (Fig. 4e, f), the configuration becomes closer to the one observed for F-and O-terminated materials.

These considerations also explain the energy value trends in Fig. 2d. All energy gains are greater than 1.00 eV, except for the 1V_Ti_ + 1V_T_ clusters on OH-terminated surfaces, where the re-orientation of hydroxyls reduces their reactivity. Furthermore, for O- and F-terminated surfaces, the energy gains related to the water chemisorption on 1V_Ti_ + *q*V_T_ clusters decrease with increasing *q* due to the loss of reactivity explained before. However, energy fluctuations may also result from the complex interplay between other factors. The average distance between the oxygen atom of water and Ti atoms, as well as the positive atomic charge on Ti atoms, are also found to affect the extent of the water chemisorption.

The chemisorption of water on 2V_Ti_ + *q*V_T_ clusters (*q* = 1, 2, 3) is regulated by almost the same mechanisms discussed above, i.e., for the 1V_Ti_ + *q*V_T_ clusters. The full discussion for these defective sites is provided in Additional file 1: Fig. S7, while only a few comparative comments are presented here. The main difference between 2V_Ti_ + *q*V_T_ and 1V_Ti_ + *q*V_T_ lies in the number of undercoordinated Ti atoms stabilized by the oxygen atom of H_2_O. In the case of 2V_Ti_ + *q*V_T_, indeed, the presence of a double V_Ti_ leaves only one titanium atom with dangling bonds and leads to smaller energy gains compare to 1V_Ti_ + *q*V_T_ (Fig. 2e). The energy gains related to the chemisorption of water for 2V_Ti_ + *q*V_T_ are still greater than 1 eV. This can be explained by the fact that even if H_2_O interacts with only one Ti atom, the H_2_O–Ti bond distance is reduced (Additional file 1: Fig. S8), becoming comparable to the typical bond distance between Ti and T_*x*_ in a defect-free layer.

### Effect of mixed terminations

**Fig. 5:**
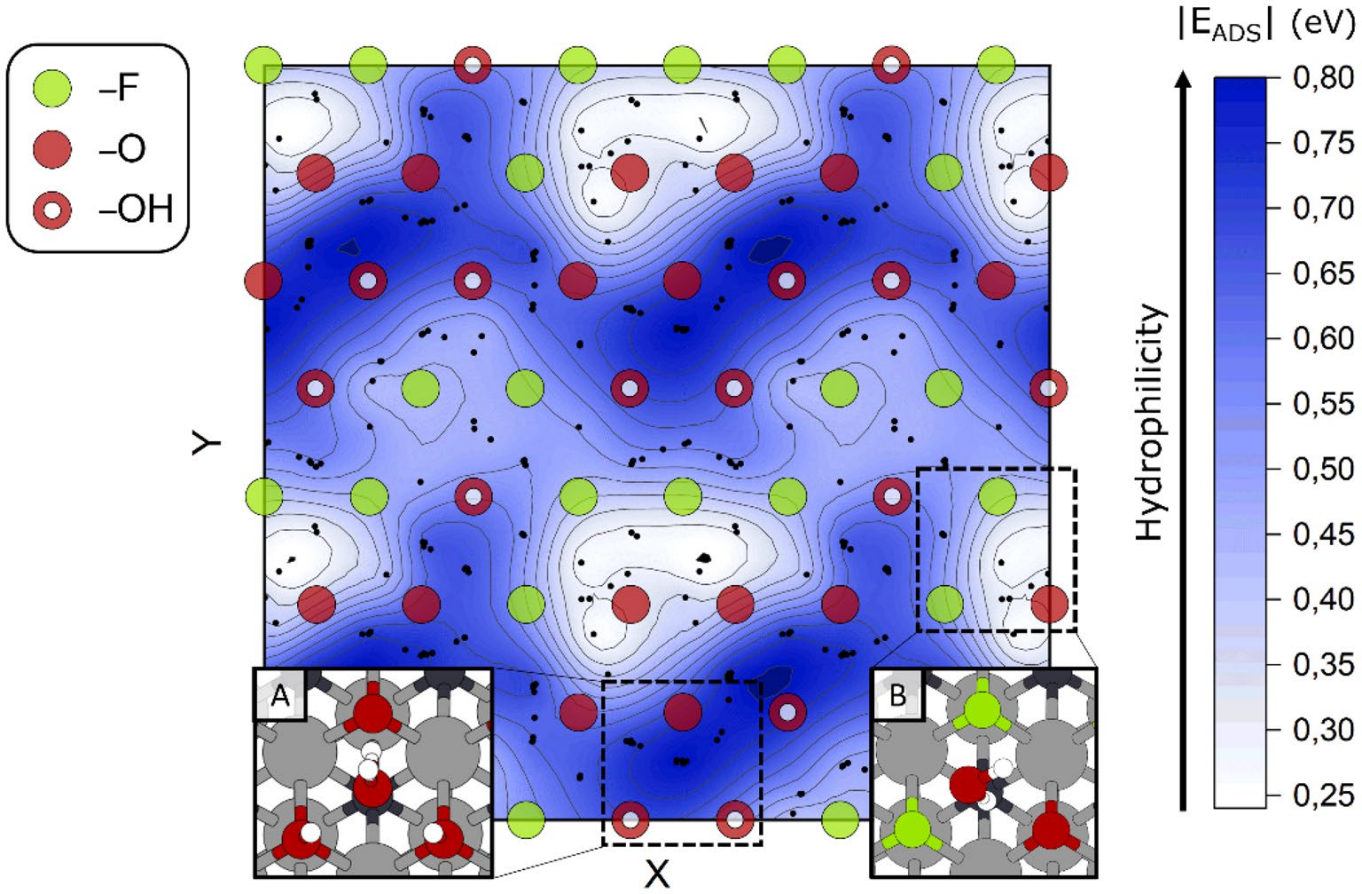
Hydrophilicity map of a 2 × 2 Ti_2_CT_*x*_ with mixed terminated surface. The two axes identify the lateral displacement on the surface, referring to the termination positions. Terminations are shown in relief on the map. Hydrophilic regions (blue) are those rich in hydroxyl groups, where hydrogen bond networks are easily formed (inset A). White regions correspond to less hydrophilic areas, where H_2_O interacts only moderately with the surface (inset B). Black tiny dots identify the average x and y coordinates of H_2_O, for each of the 96 optimized water molecules

It is known that as synthesized MXene surfaces are generally covered by a mixture of randomly distributed surface terminations [38–41]. Our group has already verified that the chemical behavior of mixed and homogeneous MXene surfaces can be quite different from each other [46]. To gain a better understanding of MXenes oxidation, we investigated the interaction of water with a Ti_2_CT_*x*_ layer simultaneously passivated with –F, –O and –OH (in a stoichiometric ratio of about one-third each). The hydrophilicity map of Ti_2_CT_*x*_ (Fig. 5), was built after performing 96 separate relaxations, trying to sample all the supercell areas in a uniform way. For each optimized configuration, we collected the final position of H_2_O (i.e., average x and y coordinates, tiny black dots in Fig. 5), as well as the associated energy gain (blue-white scale). As a result, we created the hydrophilicity map after making the interpolation on our obtained data points. The solid curved lines in black identify level curves that connect points with the same hydrophilicity (i.e., energy gain).

In the hydrophilicity map presented in Fig. 5, dark blue areas highlight strongly hydrophilic areas, while white areas identify those regions that only mildly interact with water. Terminations are superimposed on the map to better appreciate their different contribution to making a surface hydrophilic. In insets A and B, we provide two exemplary configurations, corresponding to an energy gain of 0.80 eV and 0.25 eV, respectively. The unique ability of hydroxyl groups to act as H-bond donors leads to adsorption energy gains, which are always greater than 0.50 eV. Indeed, a higher density of –OH terminations leads to more interaction with water and greater hydrophilicity. In contrast, –O and –F terminations always behave as H-bond acceptors, while oxygen is always preferred whenever possible.

These results can be compared to the case of homogeneously terminated surfaces, for which the maximum adsorption energy gain was found to be ≈ 0.50 eV for Ti_2_C(OH)_2_ and Ti_2_N(OH)_2_. Consequently, the consideration of a realistic surface having a mixture of surface terminations groups makes the system even more hydrophilic. Interestingly, the tendency to strongly interact with water is not maximized for a full coverage of hydroxyl groups. A balanced ratio between hydrogen bond donors and acceptors is the key factor in inducing cooperative hydrophilic effects. Although we limited this section to defect-free mixed terminated surfaces, we suppose that an increase in the interaction with water could arise for defective surfaces, which will be addressed in a follow-up study.

### Effect of water coverage

**Fig. 6:**
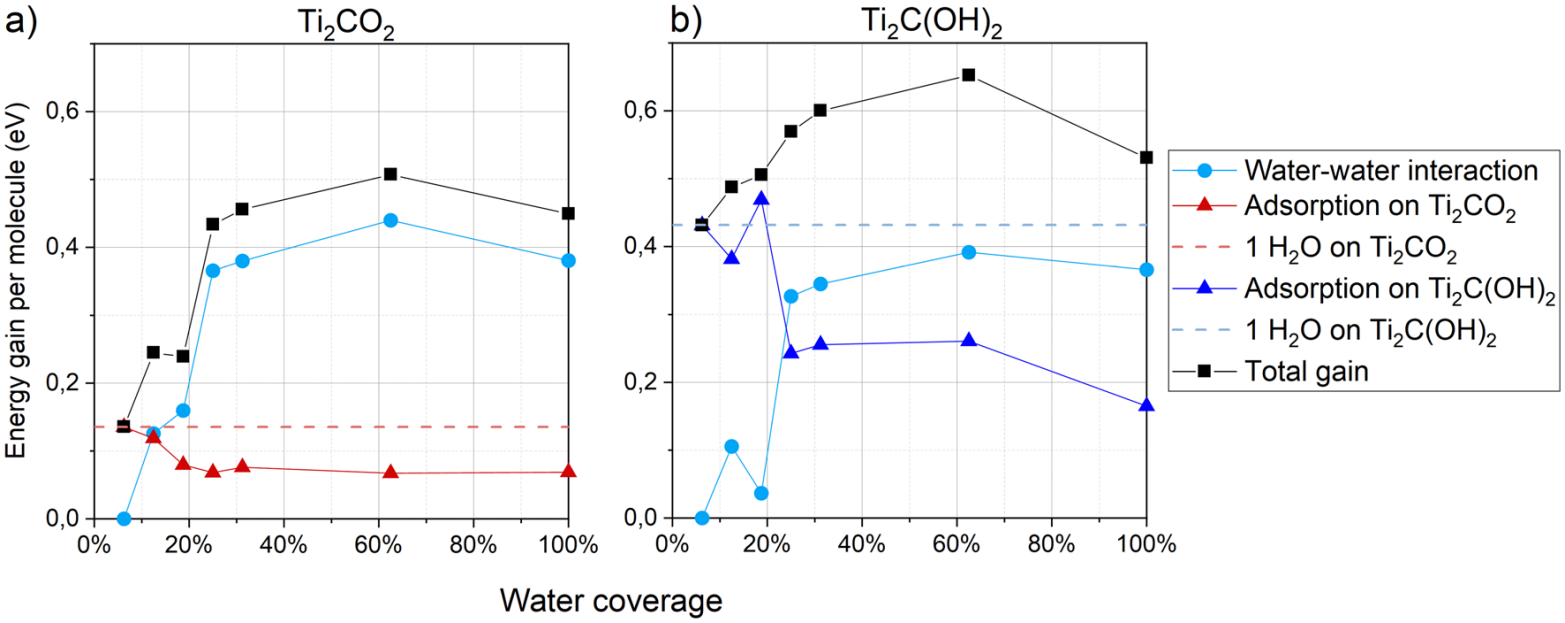
Energy gain per molecule as a function of the water coverage for **a** Ti_2_CO_2_ and **b** Ti_2_C(OH)_2_. Two contributions to the total energy (black line) are considered. The interaction between water molecules is in light blue. The interaction between the water layer and the substrate is colored depending on the type of termination. The dashed line indicates the interaction value for a single water molecule, corresponding to a coverage of 6%. Solid lines between points are guides to the eye

In this section, we discuss the effect of an increased water coverage from 6 to 100% for defect-free surfaces. Energy gains per water molecule are indicated with black squares in Fig. 6 for two types of Ti_2_CT_*x*_, namely Ti_2_CO_2_ and Ti_2_C(OH)_2_. The other MXene compositions that we studied here, Ti_2_CF_2_, Ti_4_C_3_F_2_ Ti_2_NF_2_, and Ti_2_C(OH)_2_ are similar to the two examples shown here (Ti_2_CO_2_ and Ti_2_C(OH)_2_) as presented in Additional file 1: Fig. S9. We decided to split the total energy gain per water molecule into two additive contributions: one arising from the interaction exclusively between H_2_O molecules (light blue spheres), and the other that considers the pure interaction of the water layer with the surface (red and blue triangles).

Regardless of the surface composition, the total energy gain (black squares) increases until it reaches a maximum value at ~ 62.5% coverage. This trend arises from the sum of the two separate contributions mentioned before. The interaction of the water layer with the surface, triangles in Fig. 6, tends to decrease while increasing the H_2_O coverage. At the same time, increasing the water coverage also produces a growth in the interaction between H_2_O molecules within the water layer (light blue dots), that reaches an asymptotic value of about 0.38 eV. This value can be traced back to the interaction energy resulting from a strong H-bonds network, which does not depend on the type of termination. Comparing the total energy gain in Fig. 6a with Fig. 6b once again suggests the higher hydrophilicity induced by –OH compared to –O groups (and –F groups in Additional file 1: Fig. S9). The ability of hydroxyl groups to act as H-bond donors is the key factor, which is reflected in increased interaction between the surface and water layer.

**Fig. 7:**
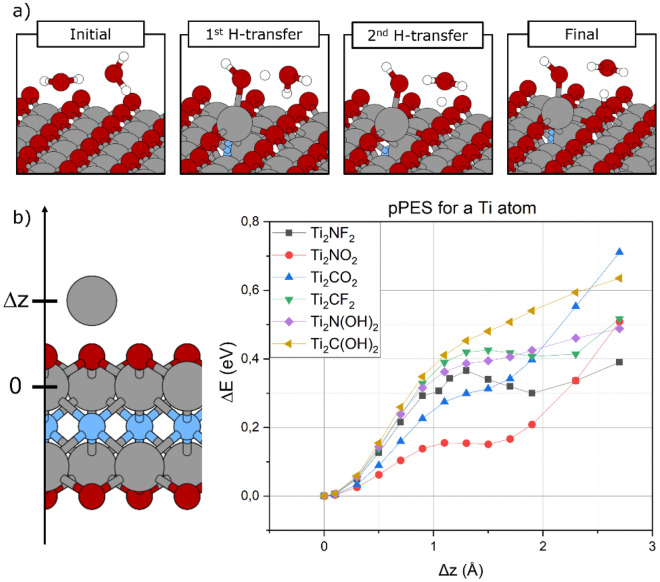
Relevant steps for the Ti_2_NO_2_ degradation caused by the presence of water (**a**). Schematic explaining how the pPES calculations where performed and energy barriers related to the extraction of a Ti atom (**b**)

Ti_2_NO_2_ surface was excluded from the previous discussion as it undergoes a structural degradation, which holds true for even low water coverages. The mechanism, observed during the relaxation of the system, is depicted in Fig. 7a for a water layer composed of two molecules. The first step involves breaking the bonds between nitrogen and a Ti atom, which is extracted from the Ti atom typical reticular position to interact with the oxygen of H_2_O. At the same time, a proton is transferred from one molecule to another, and then to the MXene surface. The presence of a second water molecule is mandatory as it catalyzes the proton transfer from the first water to an oxygen atom of the surface termination. Furthermore, between the considered terminations, oxygen is the only surface group able to accept a proton.

To investigate the unusual behavior of Ti_2_NO_2_, we computed the energetic barriers for the extraction of a titanium atom. The perpendicular Potential Energy Surfaces (pPESes) for this process are shown in Fig. 7b for 6 different layers of Ti_2_XT_2_, with X = C or N, and T = F, O or OH. pPESes are obtained by changing the z coordinate of a Ti atom and recording the energy of each configuration. Interestingly, the energy barrier related to the removal of a Ti atom has the lowest value for Ti_2_NO_2_ (0.16 eV). The intrinsic weakness of the N–Ti bonds in Ti_2_NO_2_ could explain the experimental evidence that nitride MXenes are difficult to synthesize by means of conventional etching in aqueous acidic solutions [64, 65].

Under ambient conditions, the H_2_O concentration is higher than the coverages considered in this study and multilayer structures are present. However, due to the lack of previous studies in literature, we consider worth starting to build some knowledge on molecular adsorption, in particular on the role of defects in strengthening the water-MXene interaction. The formation of multi-layer water structures may be promoted by the first chemisorbed molecules through the formation of hydrogen-bonds. Moreover, MXenes attract a lot of interest as solid lubricants [22]. Since the tribological properties of 2D materials, such as graphene and MoS_2_, are notably affected by the physical/chemical interaction of the layers with water molecules [66–68], we believe that the present study is useful to interpret the tribological behavior of MXenes measured for different relative humidities.

## Conclusions

In this work, the interaction of titanium-based MXenes with water has been characterized through static DFT calculations. The intrinsic hydrophilicity of defect-free surfaces has been explored depending on the type of termination, carbon/nitrogen in X, and MXene layer thickness (*n*). Subsequently, we have unraveled the role of single atomic vacancies, cluster vacancies and stable edges in promoting the chemisorption of water. Our results suggest that H_2_O chemisorption on MXene surfaces only occurs in the presence of undercoordinated titanium atoms. The absence of at least one termination group (V_T_) resulted in a destabilization of the adjacent Ti atoms, which strongly interacted with the oxygen atom of water to saturate their dangling bonds. Without this dangling bond, water simply physically adsorbs on the substrate via the formation of hydrogen bonds with the terminations, i.e., –OH, –O and –F.

Physical adsorption occurs both on defect-free surfaces and substrates with a single vacancy of C/N (V_C/N_) or Ti atom (V_Ti_). In these cases, energy gains are influenced by two factors: the number of established H-bonds, as well as their intensity. MXenes’ surface terminations were found to be the main regulator of these parameters compared to their layer thickness and C/N substitution. Hydroxyl groups provide the strongest interaction leading to increased adsorption values (0.40–0.65 eV) due to their unique ability to act as donors of H-bonds. In contrast, fluorine and oxygen terminations can only behave as weak H-bonds acceptors, showing long bond distances. For MXenes with –O and –F terminations, the resulting in-plane interaction with H_2_O (0.10–0.30 eV) is comparable to the one observed for graphene and MoS_2_ (0.12 eV and 0.15 eV, respectively).

Moreover, we demonstrated that an increased H_2_O coverage is responsible for a reduced interaction of the water layer with the surface, as well as a growth of the interaction between H_2_O molecules within the water layer. Regardless of the surface composition, the adsorption of an additional water molecule is thermodynamically always favored.

Water chemisorption has been observed on surfaces with a single termination vacancy (V_T_), on edges, and especially on almost all defect clusters (*p*V_Ti_ + *q*V_T_, with *p* = 1, 2 and *q* = 1, 2, 3). Whenever an unsaturated titanium atom appeared on the surface, the oxygen atom of water approached the defect and chemisorbed onto it. The energy gains are higher for the chemisorption on vacancy clusters (1.00–1.80 eV) compared to edges (0.75–0.85 eV) and termination vacancies (0.60–1.20 eV). This difference relates to an additional stabilizing effect that H_2_O can provide to surfaces when at least one Ti and T atom do simultaneously miss. The lack of a titanium atom accumulates the electronic charge around the V_Ti_ defect that is mitigated when one hydrogen of H_2_O is pointed towards the hole.

Further, our results show Ti_2_NO_2_ MXene has a structural instability in presence of water, which is associated with the extraction of a Ti atom from the surface. This result clearly supports the experimental difficulties encountered in the synthesis of Ti_2_NT_*x*_ when conventional methods are employed.

Overall, this work suggests that defects on the MXene surfaces can promote the chemisorption of water, which is the first step towards degradative oxidation. However, high energy gains suggest that defect clusters promote chemisorption more than edges. Our results unravel the primary mechanisms responsible for MXenes’ oxidative degradation in aqueous environments and their experimentally observed hydrophilicity. Moreover, they pave the way for further investigations concerning the effects of humidity, water coverage, and intercalated water on the tribological performance of MXenes.

## Supplementary Information

**Additional file 1.** Supercells employed in DFT calculations; strategies for the sampling of water configurations; optimized structures of MXene surfaces; structural configurations of water interacting with all the surfaces; charge analysis; correlation between adsorption energy and H_2_O–Ti distances; dependance of the energy gain on the water coverage for all the MXene compositions.

## Data Availability

The data-sets generated and/or analyzed during the current study are available in the Tribchem website, at the link (http://tribchem.it/?page_id=1663).
