# Supplementary material for: Effect of vacancies and edges in promoting water chemisorption on titanium-based MXenes
Source: Nano Converg. 2023 Apr 1;10:16. doi: 10.1186/s40580-023-00364-8 (PMC10067785; doi:10.1186/s40580-023-00364-8)
Supplement: Supplementary file 1 — Additional file 1. Supercells employed in DFT calculations; strategies for the sampling of water configurations; optimized structures of MXene surfaces; structural configurations of water interacting with all the surfaces; charge analysis; correlation between adsorption energy and H2O–Ti distances; dependance of the energy gain on the water coverage for all the MXene compositions. [file 40580_2023_364_MOESM1_ESM.docx]

Additional file 1

**Effect of vacancies and edges in promoting water chemisorption on titanium-based MXenes**

Edoardo Marquis, Francesca Benini, Babak Anasori, Andreas Rosenkranz, and Maria Clelia Righi*

E. Marquis, F. Benini, Prof. M. C. Righi

Department of Physics and Astronomy, Alma Mater Studiorum – University of Bologna, Viale Berti Pichat 6/2, 40127, Bologna, Italy

*E-mail: clelia.righi@unibo.it

Prof. B. Anasori

School of Materials Engineering, Purdue University, West Lafayette, Indiana 47907, USA

Prof. A. Rosenkranz

Department of Chemical Engineering, Biotechnology and Materials, University of Chile, Avenida Beaucheff 851, 8370456, Santiago de Chile, Chile

**Computational Details**

MXenes 4x4 orthorhombic cells are modelled starting from hexagonal unit cells, which are exemplarily shown in **Figure S1a** (top view of Ti_2_CO_2_). To optimize the in-plane lattice parameter, ‘a’, for the hexagonal cells, we carried out multiple calculations of structural optimization for different values of ‘a’. During these relaxations, the vertical lattice parameter, ‘c’, was kept fixed to ensure a vacuum region of about 15 Å along z. An ordinary least squares regression was used to fit the energies as a function of ‘a’ with a parabolic function, thus identifying the minimum. We repeated the procedure for different wave-function cutoffs (the charge density cutoff was always set as eight times the wave-function cutoff), as reported in **Figure S1b**. The convergence test for K-points grid is also displayed in **Figure S1c** for the hexagonal unit cell. An equivalent grid was used for 4x4 orthorhombic cells, corresponding to a 3x4x1 grid.

**
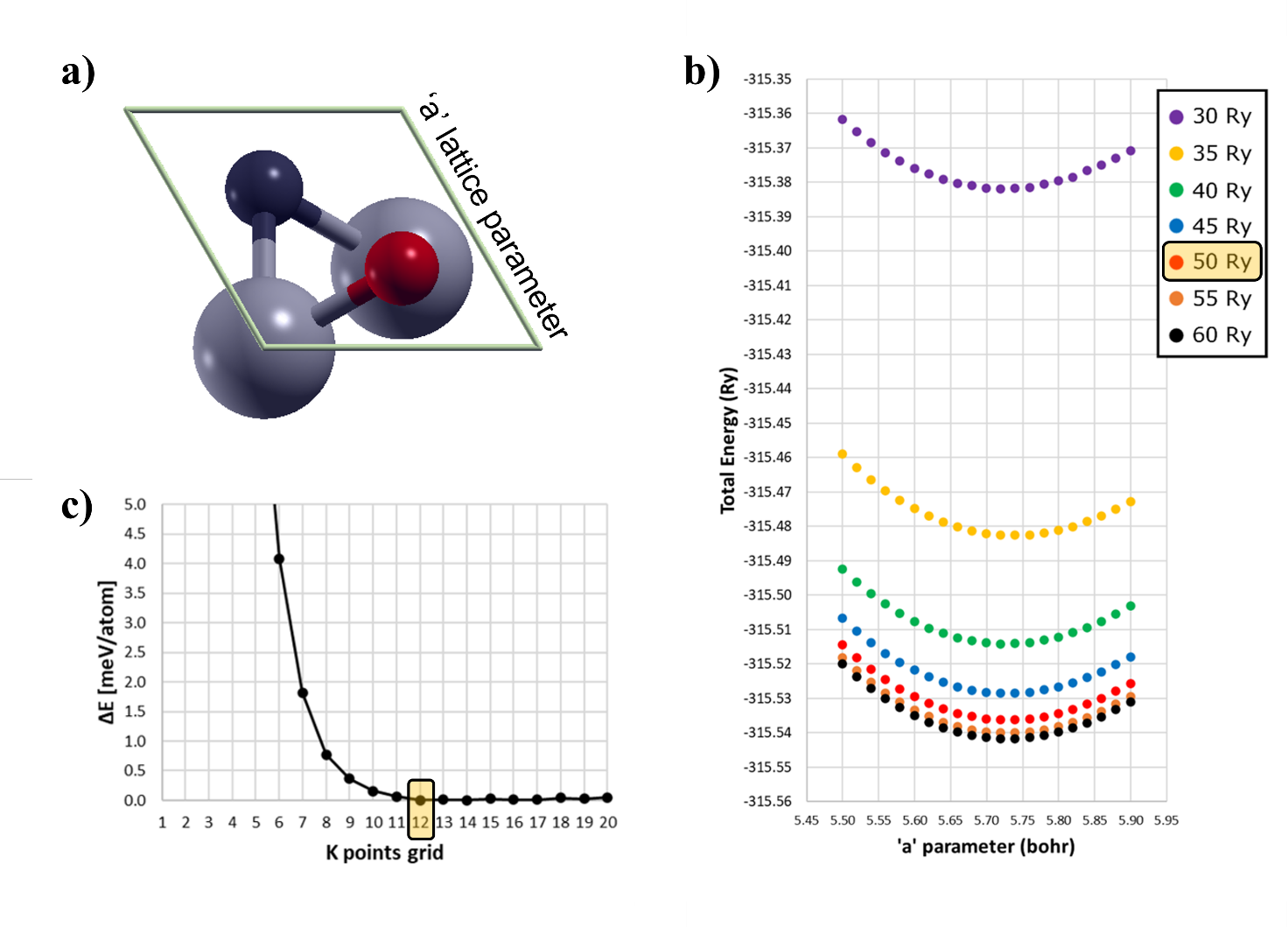
**

**Figure S1**. a) Top-view of the hexagonal unit cell for Ti_2_CO_2_ (Ti, C and O atoms are shown in grey, black and red colour, respectively). (b) Convergence test for the electronic wave-function cut-offs: the optimization of the ‘a’ lattice parameter is repeated for different cut-off values. We have chosen a plane-wave (charge density) cut-off of 50Ry (400Ry). (c) Convergence test for the NxNx1 Monkhorst-Pack grid. The convergence for the hexagonal cell is reached with a grid of 12x12x1.

**Supercells**

MXene’ orthorhombic supercells were built based on their hexagonal unit cell. In **Figure S2,** we depict an example of the 4x4 orthorhombic supercells employed in our work, corresponding to the cases of defect-free Ti_2_CF_2_ and Ti_4_C_3_F_2_. Surfaces with defects have been modelled with the same 4x4 supercell.

**
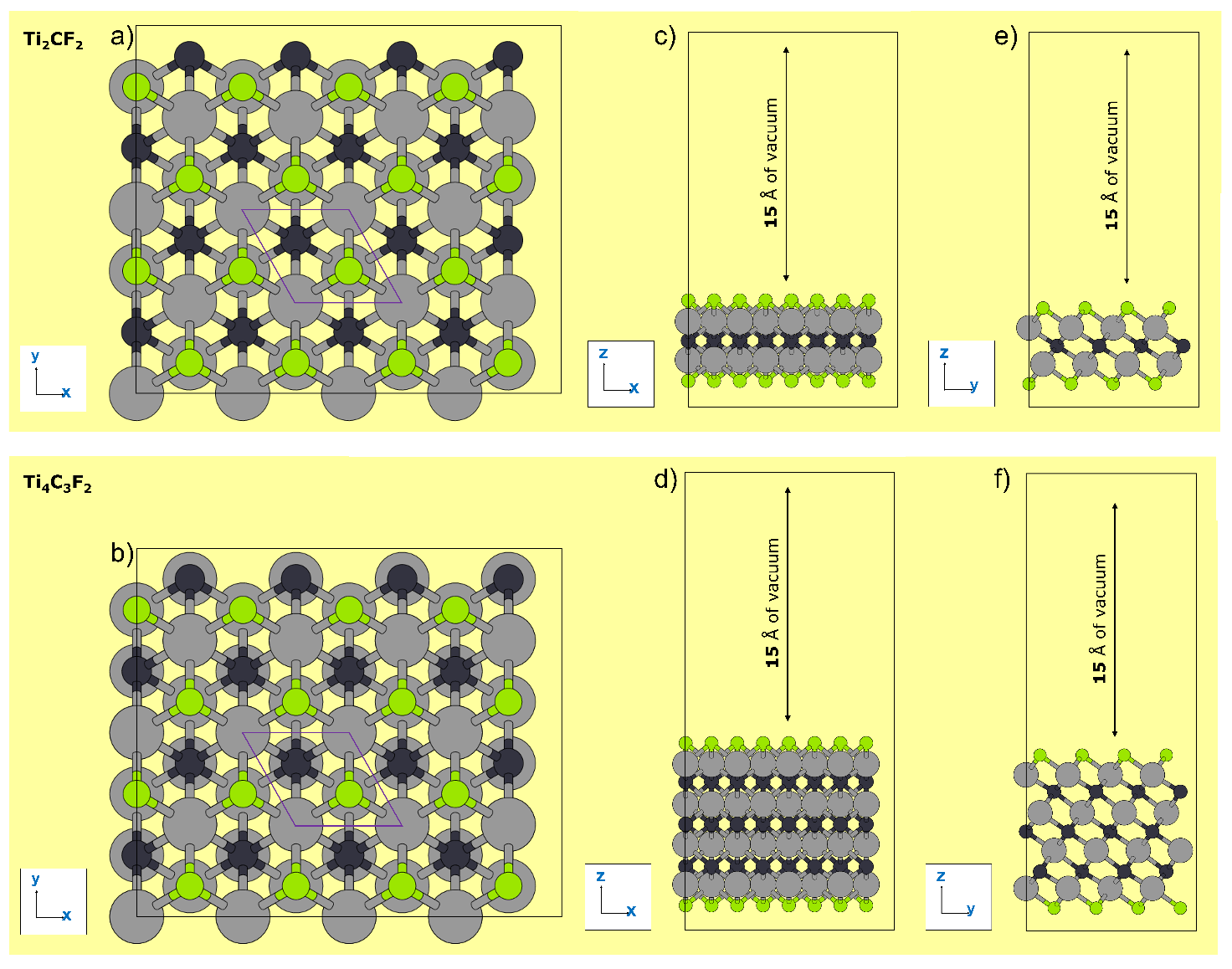
**

**Figure S2**. (a-b) Top views and (b-f) side views for the 4x4 orthorhombic supercells used for Ti_2_CF_2_ and Ti_4_C_3_F_2_. The original hexagonal unit cell is indicated in purple in the top view (left).

MXene nanoribbons were built starting from the 4x4 orthorhombic supercells by doubling the length of the *b* lattice parameter (along the y axis). The supercell employed is depicted in **Figure S3** for the case of Ti_8_N_3_(OH)_8_.

**
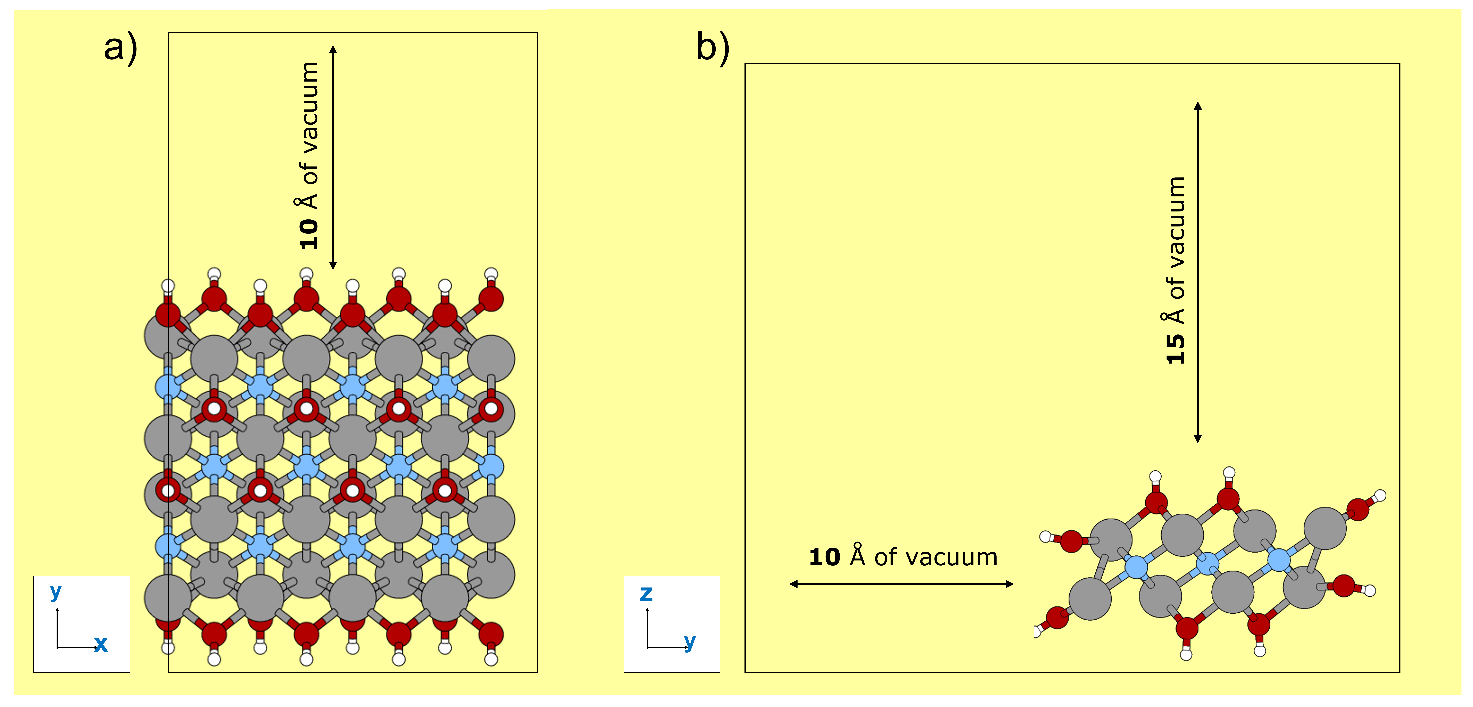
**

**Figure S3**. (a) Top and (b) side view of 1D Ti_8_N_3_(OH)_8_. The ribbon grows along the x direction. Vacuum is required along y and z axes to avoid the lateral interactions between replicas.

**Sampling of water configurations**

To explore different adsorption minima, several initial configurations concerning the orientation of water were considered for each substrate, as exemplarily shown in **Figure S4** for V_Ti_ on Ti_2_CF_2_.


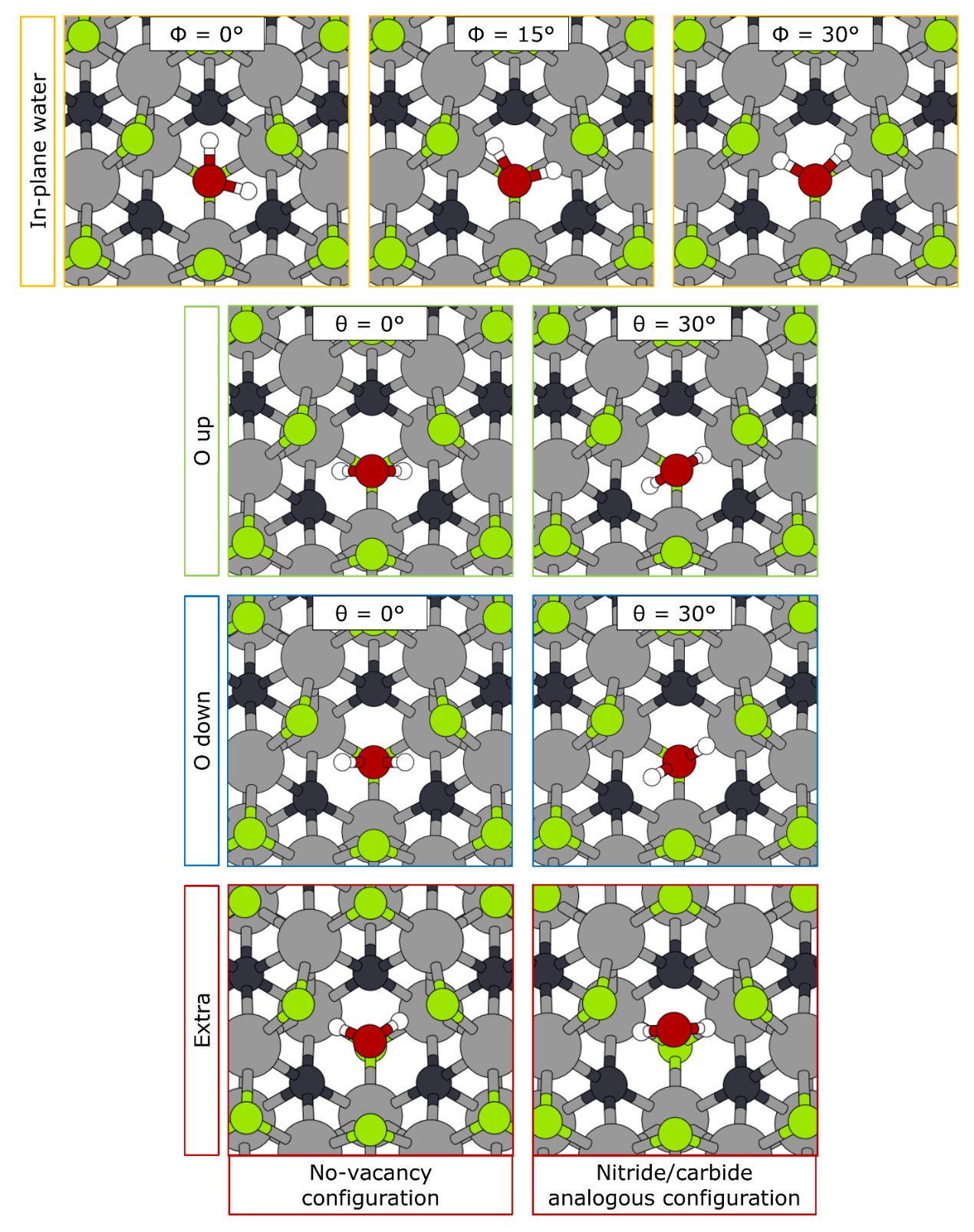


**Figure S4**. Input geometries of H_2_O explored to find the absolute minimum. Ti_2_CF_2_ surfaces with a missing Ti atom are exemplarily considered. However, it is important to point out that the same procedure has been adopted for all cases. We tried different water orientations such as in-plane, oxygen-up and oxygen-down. Rotations of the water molecule have also been tested, considering the internal symmetries of the surface (0° < θ < 30°). In addition, two extra H_2_O configurations have been tried: the one obtained from the H_2_O optimization on the full-terminated surface, and the one from the optimization of H_2_O on the analogous nitride (or carbide).

**Configurations of water physically adsorbed on surface with V_C/N_ and V_Ti_**

In **Figure S5,** we provide the relaxed configurations for H_2_O interacting with defective Ti_2_CO_2_ and Ti_2_C(OH)_2_, which have not been included in the main manuscript. Ti_2_CO_2_ is also representative for the nitride and F-terminated analogs. In contrast, Ti_2_C(OH)_2_ is representative for Ti_2_N(OH)_2_.


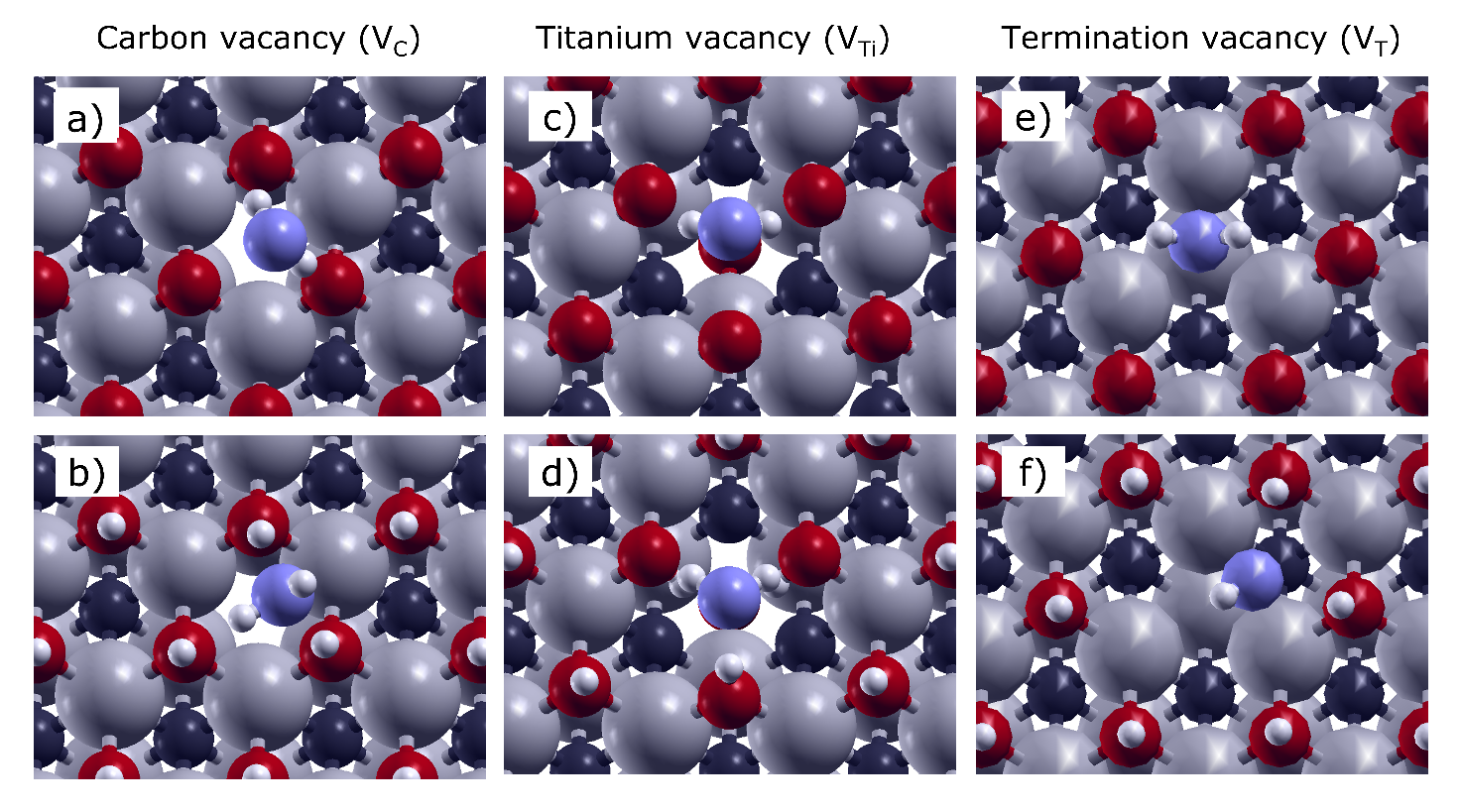


**Figure S5.** Top view of the relaxed configuration of H_2_O on surfaces with a single atom vacancy: (a-b) carbon vacancy, (c-d) titanium vacancy, (e-f) termination vacancy. (a, c and e) Ti_2_CO_2_ and (b, d and f) Ti_2_C(OH)_2_ are taken as examples. The oxygen atom of the water molecule is depicted in purple color to differentiate it from the other O atoms on the surface (in red). The interaction between water and defective surface occurs via H-bonds, apart from the case of Ti_2_CO_2_ with a termination missing (e), as discussed in the main manuscript.

**Nanoribbons and H_2_O-edge configurations**

In **Figure S6,** we provide the relaxed, cross-sectional configurations of the nanoribbons discussed in the main manuscript, as well as the top view of H_2_O interacting with the edges.


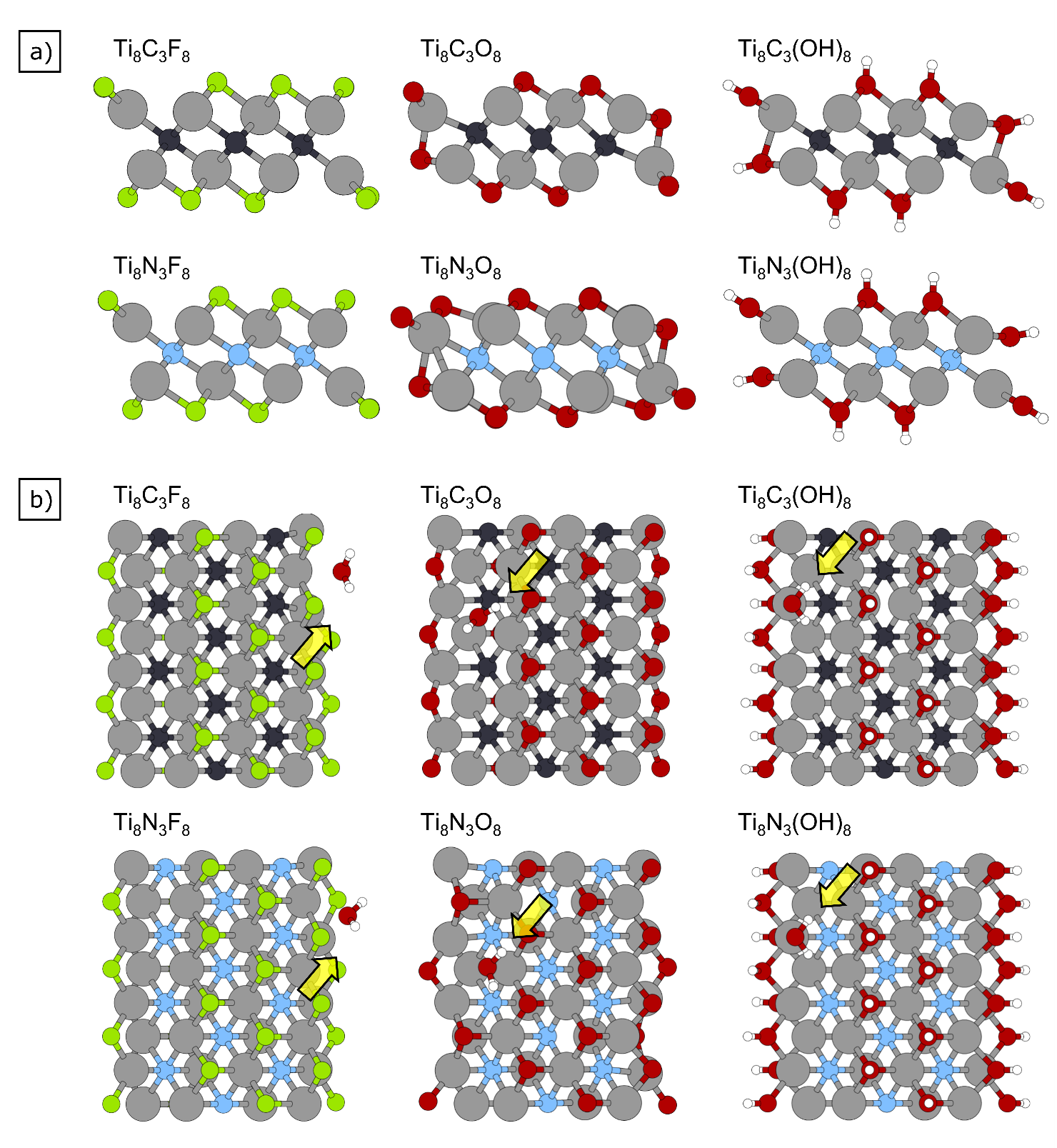


**Figure S6**. (a) Cross-sectional view of the Ti_8_X_3_T_8_ nanoribbons and (b) top view of the chemisorbed water on nanoribbons, with X = C or N, and T = F, O or OH. The transparent arrow in yellow highlights the presence of the water molecule.

**Configurations of water chemisorbed on surface with cluster of vacancies 2V_Ti_ + qV_T_ with q = 1, 2, 3**

In **Figure S7**, we present the relaxed configurations of H_2_O interacting with 2V_Ti_+*q*V_T_ (*q* = 1, 2, 3) defect clusters on Ti_2_CF_2_ and Ti_2_C(OH)_2_. The chemisorption of water on 2V_Ti_+*q*V_T_ clusters (*q* = 1,2,3) is regulated by almost the same mechanisms discussed in the main manuscript for 1V_Ti_+*q*V_T_ clusters. The most relevant difference is the number of Ti atoms interacting with the oxygen atom of H_2_O (i.e., stabilized by the oxygen of H_2_O). For instance, the presence of a double V_Ti_ in the cluster 2V_Ti_+1V_T_ (Figure S6 a-b) leaves only one titanium atom with dangling bonds (indicated with a tiny yellow arrow in Figure S6). Apart from these structural differences, energy gains related to the chemisorption of water are always greater than 1eV.


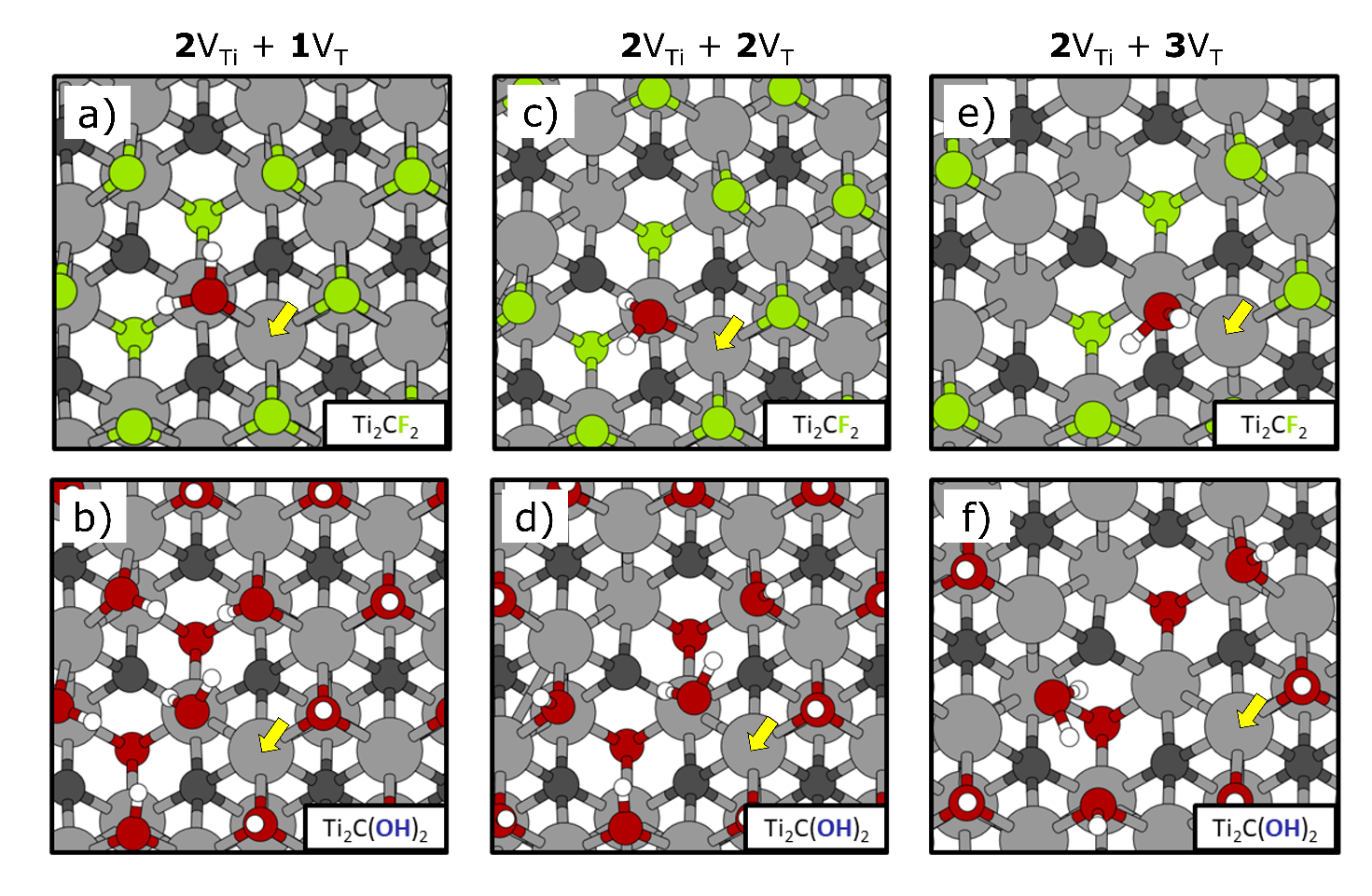


**Figure S7**. Top view for the optimized configurations of H_2_O chemisorbed on (a, c and e) Ti_2_CF_2_ and (b, d and f) Ti_2_C(OH)_2_ with 2V_Ti_ + qV_T_ (with q=1, 2, 3) vacancy clusters. The oxygen of the water molecule interacts with the remaining undercoordinated Ti atom highlighted by a tiny yellow arrow, apart from panel f, for which it stabilizes two Ti atoms instead of one.

**Energy gain VS H_2_O---Ti distances**

For 1V_Ti_+*q*V_T_ (with *m* = 1,2,3) and 2V_Ti_+1V_T_ clusters, the water molecule interacts similarly with all the substrates, regardless of the termination type. The oxygen atom of H_2_O saturates a V_T_, interacting with one/two remaining Ti atom(s), while one hydrogen atom of H_2_O is pointed towards the hole left by V_Ti_. Ti_2_C(OH)_2_ and Ti_2_N(OH)_2_ are exceptions for the cluster of defects 1V_Ti_+1V_T_, as mentioned in the main manuscript in section 3.5. We found a correlation between the energy gain related to water chemisorption and the average distance between the oxygen of H_2_O and the two Ti atoms (**Figure S8**).

Moreover, the average bond distance between H_2_O and the involved Ti atom is found to be strongly reduced for chemisorption on 2V_Ti_+1V_T_ clusters, comparable to the typical bond distance between Ti and T in a defect-free layer. The lilac squares on the left in Figure S7a, which relate to 2V_Ti_+1V_T_ clusters, are shifted towards shorter H2O---Ti distances.


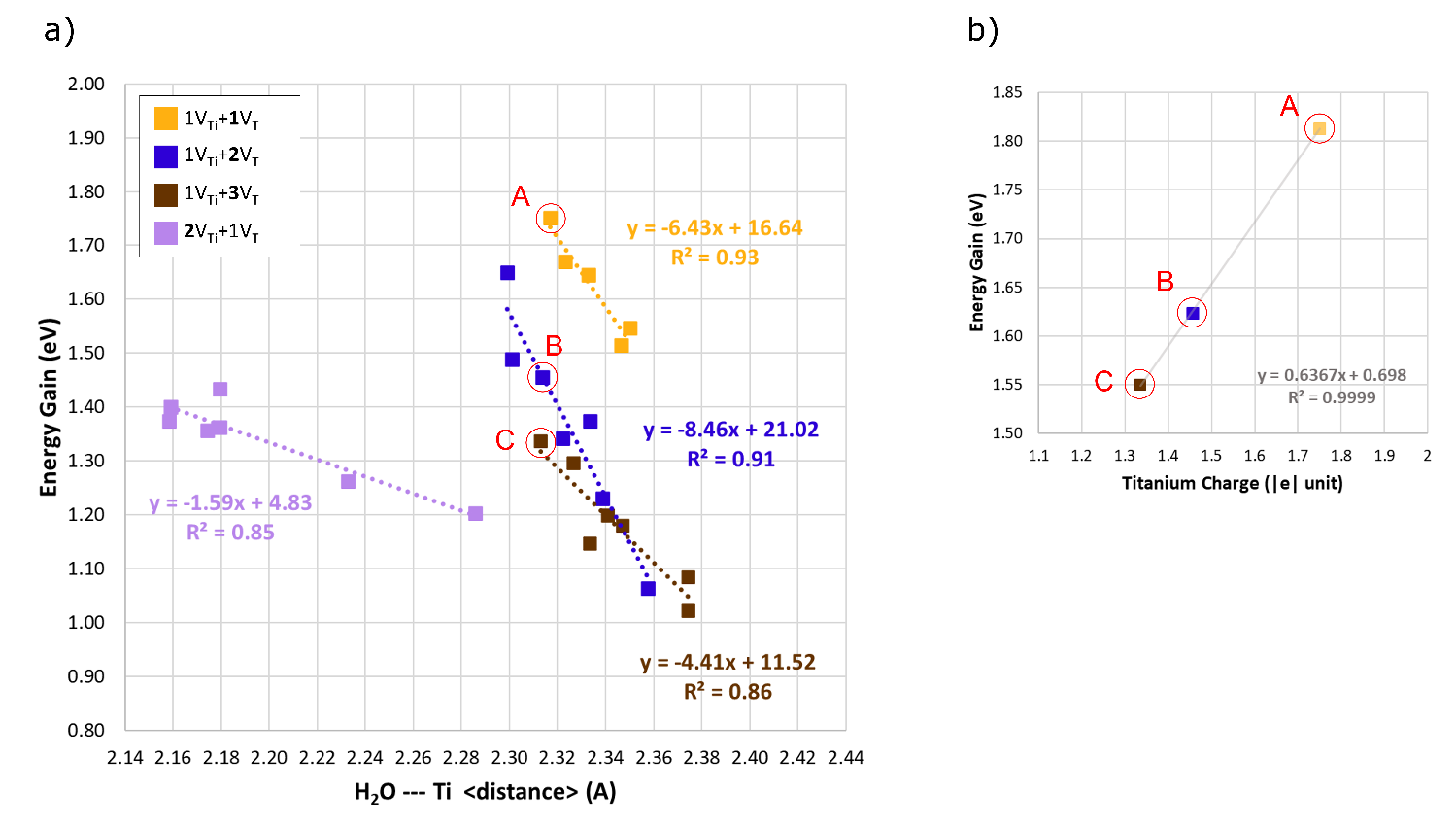


**Figure S8**. a) Energy gain versus average distance between the oxygen of H_2_O and two Ti atoms. The linear regressions highlight the correlations. Three points showing the same “H_2_O --- Ti <distance>”, while owning to different clusters of defects (1V_Ti_+1V_T_, 1V_Ti_+2V_T_ and 1V_Ti_+3V_T_), have been selected. For these cases, we calculated the partial atomic charges of the Ti atoms involved in the interaction with water, and we correlated with the corresponding energy gains (inset b). The higher the charge of Ti, the greater is the energy gain.

**Effect of water coverage for Ti_2_CF_2_, Ti_4_C_3_F_2_, Ti_2_NF_2_, and Ti_2_N(OH)_2_**

The dependance of the energy gain on the water coverage from 6% to 100% is reported in **Figure S9** for all the considered defect-free surfaces. Ti_2_NO_2_ is excluded as its reactivity towards water leads to structural degradation.


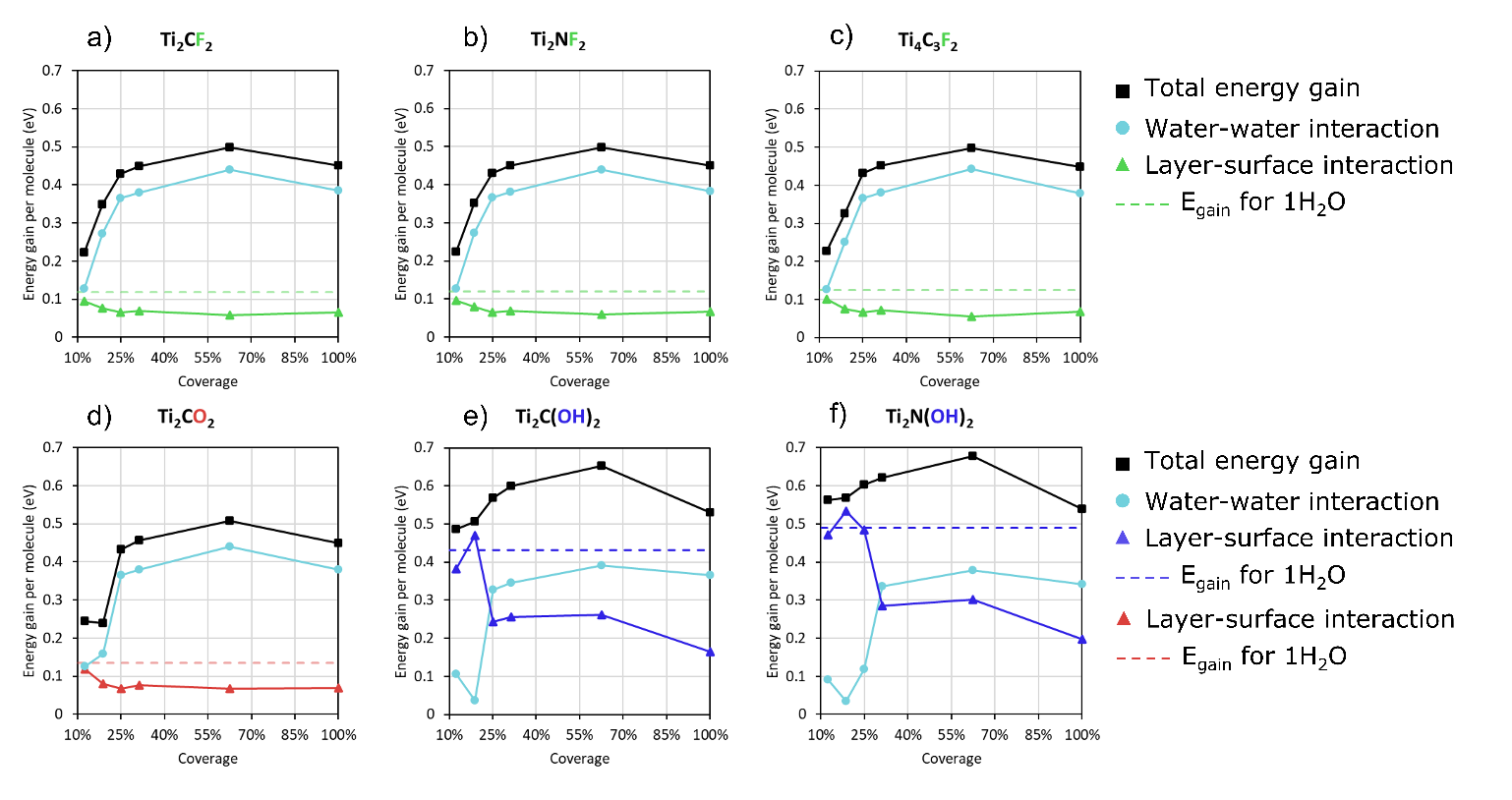


**Figure S9**. Energy gain per molecule as a function of the water coverage for (a) Ti_2_CF_2_, (b) Ti_2_NF_2_, (c) Ti_4_C_3_F_2_, (d) Ti_2_CO_2_, (e) Ti_2_C(OH)_2_ and (f) Ti_2_C(OH)_2_. Both contributions (water-water and layer-surface interaction) to the total energy (black line) are separately shown. The interaction between the water molecules is highlighted in light blue. The interaction between the water layer and the substrate is colored depending on the type of termination. The dashed line indicates the interaction value for a single water molecule, corresponding to a coverage of 6%. O- and F-terminated MXenes behave almost identically, while OH-terminated carbide and nitride show a similar trend as well.

**Charge analysis**

The correlation between the energy gain related to H_2_O chemisorption and the residual partial atomic charge on titanium have been discussed in the main manuscript for V_T_ substrates. In **Figure S10,** we collect the calculated Bader charges on V_T_ substrates.


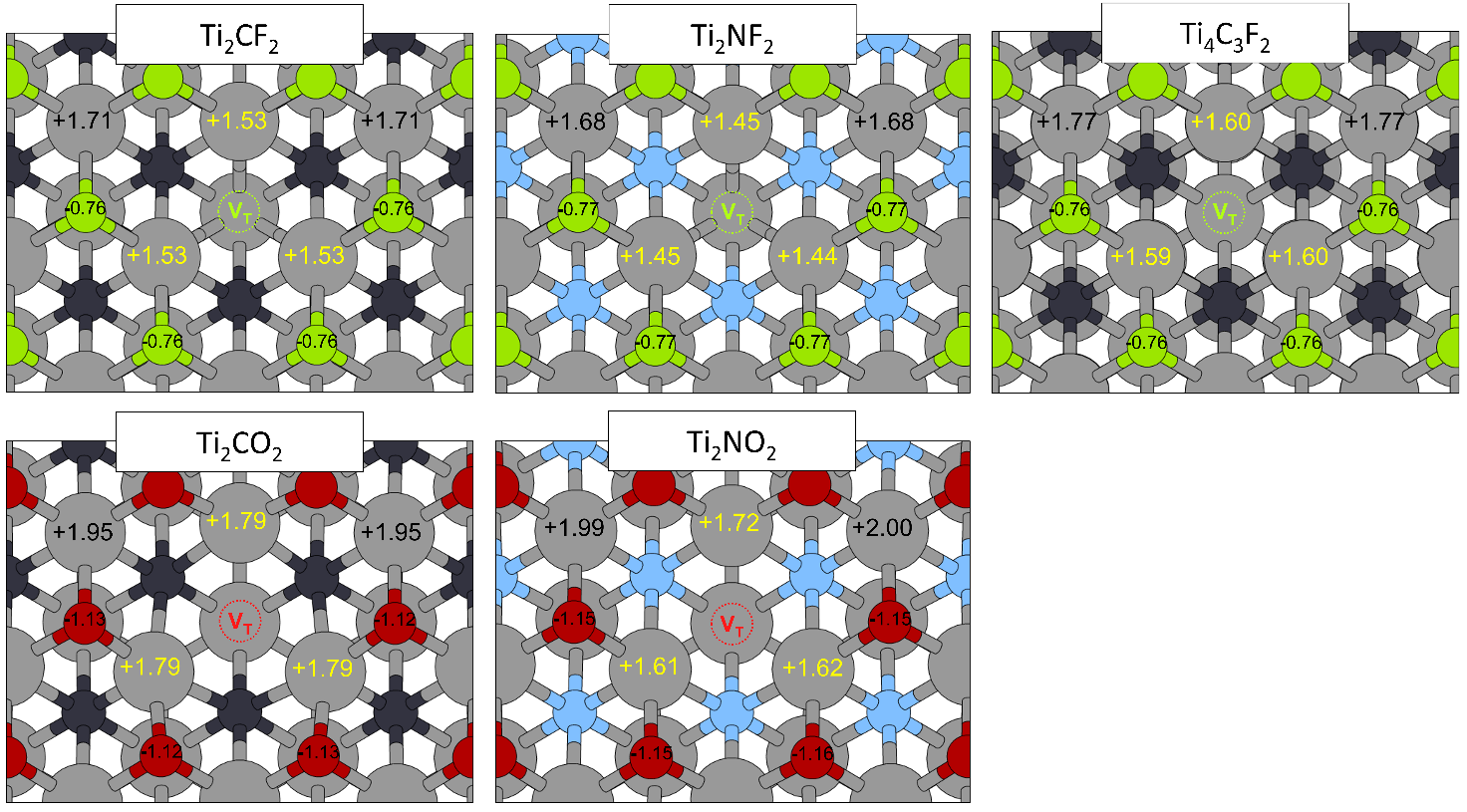


**Figure S10**. Calculated Bader charges are indicated on the –O/–F terminations and Ti atoms surrounding the vacancy of termination (V_T_). The three titanium atoms close to the vacancy show a lower positive charge than the defect-free case.

**Optimized structures of MXene substrates**

A selection of relaxed MXene substrates employed in our work is reported in **Figure S11**, **Figure S12** and **Figure S13**.


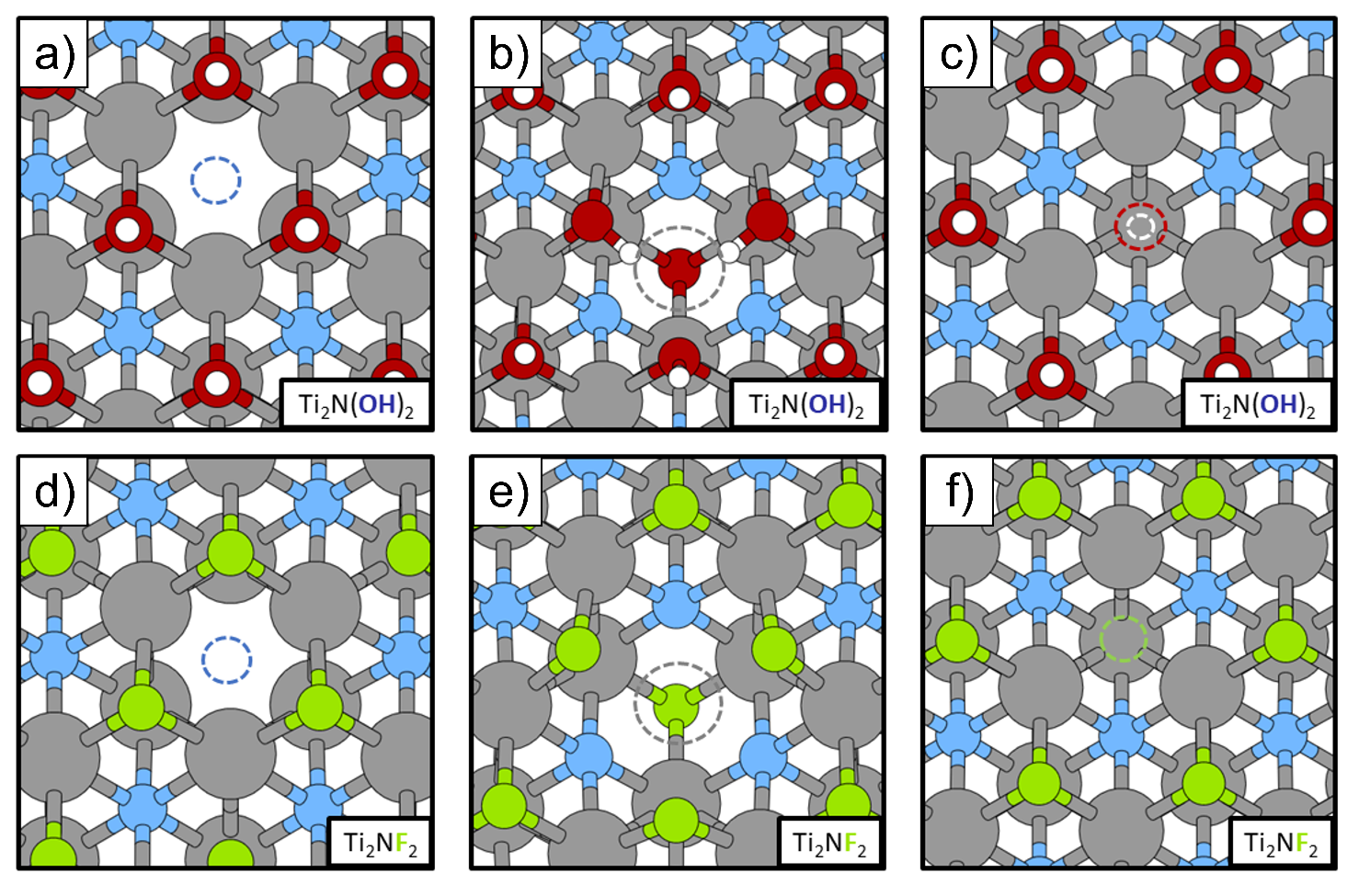


**Figure S11**. Top view of relaxed surfaces with a single vacancy for (a-c) Ti_2_N(OH)_2_ and (d-f) Ti_2_NF_2_. (a and d) Single vacancy of one N atom (V_N_). (b and e) Single vacancy of one Ti atom (V_Ti_). (c and f) Single vacancy of a termination (V_T_). Missing atoms are indicated with dashed circles. A surface reconstruction is observed only for V_Ti_ (b and e), consisting of a displacement of the surrounding terminating groups that move closer to the two remaining Ti atoms to interact more strongly. For the case of a V_Ti_ vacancy on the OH-terminated surface (b), hydroxyl groups can rotate to point their hydrogen atom towards the Ti vacancy.


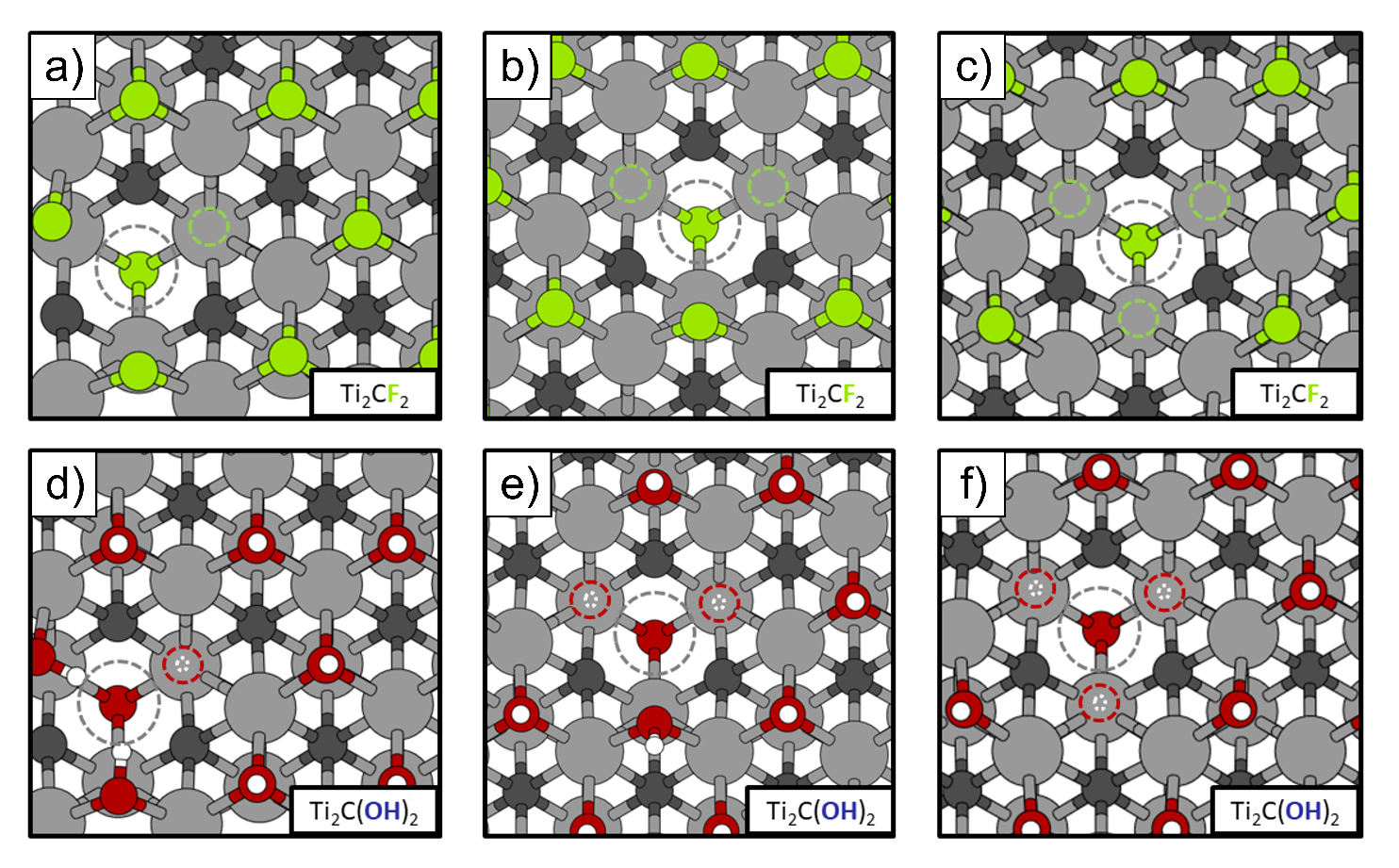


**Figure S12**. Relaxed surfaces with 1V_Ti_ + qV_T_ (with m=1,2,3) clusters of vacancies for (a-c) Ti_2_CF_2_ and (d-f) Ti_2_C(OH)_2_. (a and d) 1V_Ti_ + 1V_T_ cluster; (b and e) 1V_Ti_ + 2V_T_ cluster; (c and f) 1V_Ti_ + 3V_T_ cluster. Missing atoms are indicated with dashed circles. Due to the hole caused by the V_Ti_, the surrounding terminating groups move closer to the remaining Ti atoms to interact more strongly. For OH-terminated surfaces (d-f), the remaining hydroxyls can rotate to point their hydrogen atom towards the Ti vacancy, providing a stabilization to the defect.


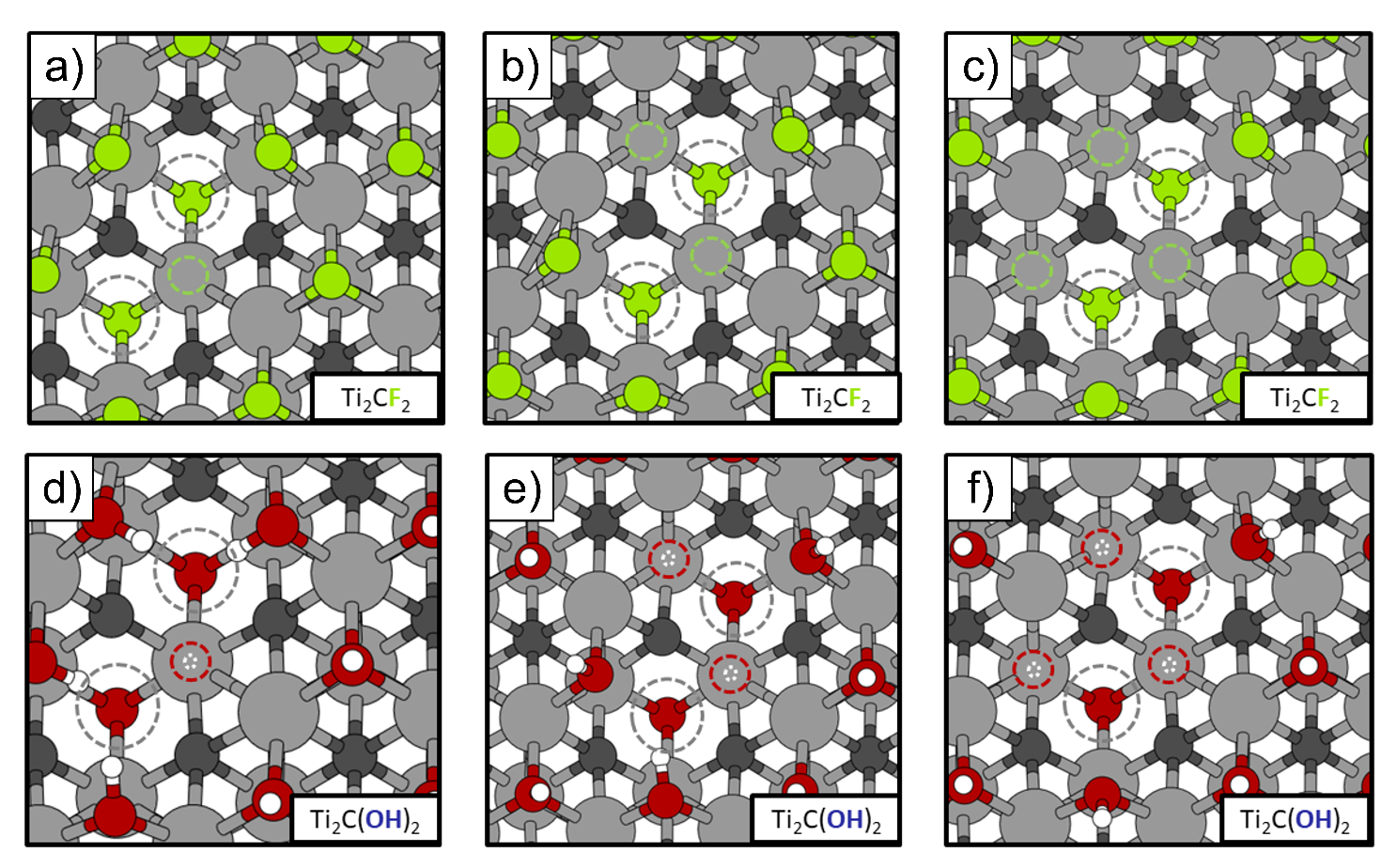


**Figure S13**. Relaxed substrates with 2V_Ti_ + qV_T_ (with m=1,2,3) clusters of vacancies for (a-c) Ti_2_CF_2_ and (d-f) Ti_2_C(OH)_2_. (a and d) 2V_Ti_ + 1V_T_ cluster; (b and e) 2V_Ti_ + 2V_T_ cluster; (c and f) 2V_Ti_ + 3V_T_ cluster. Missing atoms are indicated with dashed circles. Surface reconstruction is similar to Figure S11. The lack of Ti atoms causes the surrounding terminations to move away from their lattice position, to increase the interaction with the remaining titanium atoms. The -OH groups (d-f) can rotate to stabilize the surface, pointing their hydrogen atom towards any accumulation of charge.
